# S2e guideline: positioning and early mobilisation in prophylaxis or therapy of pulmonary disorders: Revision 2015: S2e guideline of the German Society of Anaesthesiology and Intensive Care Medicine (DGAI)

**DOI:** 10.1007/s00101-015-0071-1

**Published:** 2015-09-03

**Authors:** Th. Bein, M. Bischoff, U. Brückner, K. Gebhardt, D. Henzler, C. Hermes, K. Lewandowski, M. Max, M. Nothacker, Th. Staudinger, M. Tryba, S. Weber-Carstens, H. Wrigge

**Affiliations:** Clinic for Anaesthesiology, University Hospital Regensburg, 93042 Regensburg, Germany; Physiotherapy Department, Clinic Donaustauf, Centre for Pneumology, 93093 Donaustauf, Germany; Clinic for Anaesthesiology, Surgical Intensive Care Medicine, Emergency Care Medicine, Pain Management, Klinikum Herford, 32049 Herford, Germany; HELIOS Clinic Siegburg, 53721 Siegburg, Germany; Clinic for Anaesthesiology, Intensive Care Medicine and Pain Management, Elisabeth Hospital Essen, 45138 Essen, Germany; Centre Hospitalier, Soins Intensifs Polyvalents, 1210 Luxembourg, Luxemburg; Association of Scientific Medical Societies (AWMF), 35043 Marburg, Germany; University Hospital for Internal Medicine I, Medical University of Wien, General Hospital of Vienna, 1090 Vienna, Austria; Clinic for Anaesthesiology, Intensive Care Medicine and Pain Management, Klinikum Kassel, 34125 Kassel, Germany; Clinic for Anaesthesiology and Surgical Intensive Care Medicine, Charité Universitätsmedizin Berlin, Campus Virchow Klinikum, 13353 Berlin, Germany; Clinic and Policlinic for Anaesthesiology and Intensive Care Medicine, University Hospital Leipzig, 04103 Leipzig, Germany

**Keywords:** positioning therapy, early mobilisation, prone position, pulmonary disorder, backrest elevation, continuous lateral rotation, Lagerungstherapie, Frühmobilisation, Bauchlagerung, Pulmonale Funktionsstörung, Oberkörper Hochlagerung, Kontinuierliche laterale Rotationstherapie

## Abstract

The German Society of Anesthesiology and Intensive Care Medicine (DGAI) commissioneda revision of the S2 guidelines on “positioning therapy for prophylaxis or therapy of pulmonary function disorders” from 2008. Because of the increasing clinical and scientificrelevance the guidelines were extended to include the issue of “early mobilization”and the following main topics are therefore included: use of positioning therapy and earlymobilization for prophylaxis and therapy of pulmonary function disorders, undesired effects and complications of positioning therapy and early mobilization as well as practical aspects of the use of positioning therapy and early mobilization. These guidelines are the result of a systematic literature search and the subsequent critical evaluation of the evidence with scientific methods. The methodological approach for the process of development of the guidelines followed the requirements of evidence-based medicine, as defined as the standard by the Association of the Scientific Medical Societies in Germany. Recently published articles after 2005 were examined with respect to positioning therapy and the recently accepted aspect of early mobilization incorporates all literature published up to June 2014.

## Zusammenfassung

Durch die Deutsche Gesellschaft für Anästhesiologie und Intensivmedizin (DGAI) wurde der Auftrag erteilt, die seit 2008 bestehende S2-Leitlinie „Lagerungstherapie zur Prophylaxe oder Therapie von pulmonalen Funktionsstörungen“ zu revidieren. Aufgrund zunehmender klinischer und wissenschaftlicher Relevanz wurde die Leitlinie um den Themenkomplex „Frühmobilisation“ erweitert. Damit bezieht sie sich auf folgende thematische Schwerpunkte: Einsatz von Lagerungstherapie und Frühmobilisation zur Prophylaxe pulmonalerFunktionsstörungen, Einsatz von Lagerungstherapie und Frühmobilisation zur Therapie pulmonaler Funktionsstörungen, unerwünschte Wirkungen und Komplikationen von Lagerungstherapie und Frühmobilisation sowie praktische Aspekte beim Einsatz von Lagerungstherapie und Frühmobilisation. Diese Leitlinie ist das Ergebnis einersystematischen Literaturrecherche sowie der anschließenden kritischen Evidenzbewertungmit wissenschaftlichen Methoden. Das methodische Vorgehen des Leitlinienentwicklungsprozesses entspricht den Anforderungen an die evidenzbasierte Medizin, wie sie von der Arbeitsgemeinschaft der Wissenschaftlichen Medizinischen Fachgesellschaften als Standard definiert wurden. Bezüglich der Lagerungstherapie wurden neu publizierte Arbeiten ab 2005 untersucht; der neu aufgenommene Aspekt der Frühmobilisation umfasst die gesamte bisher publizierte Literatur bis einschließlich 06/2014. Der vorliegende Beitrag gibt die Kurzversion der Leitlinie wieder.

## Preface

The order to revise the S2 guideline ‘positioning in prophylaxis or therapy of pulmonary disorders’, which was established in 2008, was issued by the German Society for Anaesthesiology and Intensive Care Medicine (DGAI). Due to increasing clinical and scientific relevance, the guideline was expanded to include the topic area ‘early mobilisation’.

‘Guidelines are systematically developed presentations and recommendations with the purpose of assisting physicians and patients in deciding on appropriate measures for medical care (prevention, diagnostics, therapy and after care) under specific medical conditions.’ (Association of Scientific Medical Societies, AWMF).

The guideline is based on the following fundamental assumptions:

- Guidelines for use in positioning therapy and early mobilisation in prophylaxis or therapy of pulmonary disorders aid in decision-making in specific situations. They are based on the current state of scientific knowledge and on procedures proven in practice.
- Positioning and early mobilisation are supporting concepts in the treatment and prophylaxis of pulmonary disorders, wherein they are intended to supplement basic medical measures (e.g. mechanical ventilation, fluid management, pharmacotherapy), but not to replace them.
- There is no single ‘ideal’ position for all pulmonary disorders; rather the positioning plan must be customised individually to the circumstances surrounding a patient and condition.
- A sharp distinction of the indication ‘prophylaxis’ versus ‘therapy’ is not possible for all eligible pulmonary disorders. As in other therapeutic fields, there is frequently a smooth transition between ‘prophylaxis’, ‘early treatment’ and ‘therapy’.
- On the basis of the present guideline, the majority of patients with pulmonary disorders should respond well to therapy in conjunction with a whole therapeutic plan.
- Effective teamwork, the introduction of practical algorithms and proper management of emergency situations are the requirement for the safe implementation of positioning methods and, in particular, for early mobilisation. In doing so, the integration of these concepts into everyday work procedures will lead to a routine course of action and increased experience.
- The use of positioning and early mobilisation throughout the duration of therapy requires the continual critical review of the indication and customisation to the individual progression of the disease.
- Objectives and methods of the treatment plan must be presented in a transparent manner for all involved (physicians, caregivers, physical therapists, relatives and, to the extent possible, the patient).

## Guideline topics

The guideline refers to the following topics of focus:

- The use of positioning and early mobilisation in prophylaxis of pulmonary disorders.
- The use of positioning and early mobilisation in treating pulmonary disorders.
- Undesired effects and complications of positioning and early mobilisation.
- Practical aspects when using positioning and early mobilisation.

The statements made in the guideline with respect to acute respiratory distress syndrome (ARDS) refer to the ‘Berlin definition’ [90]. This includes the following criteria for the diagnosis of ARDS:

- **Begin**: within a week after an acute incident or recently occurred or worsened symptoms
- **Imaging** (X-ray or computed tomography (CT) scan of chest): bilateral infiltrations that cannot be explained alone by effusion, pneumothorax or nodules
- **Cause** of the oedema: respiratory distress cannot be explained alone through acute heart failure or volume overload (in the case of a lack of risk factors, the presence of hydrostatic oedema by means of echocardiogram must not be precluded)
- **Oxygenation:** three degrees of severity are differentiated
  **mild**: partial arterial pressure of oxygen (PaO_2_)/fractional inspiratory concentration of oxygen (FIO_2_)= 200–299 mm Hg and positive end-expiratory pressure (PEEP)/continuous positive airway pressure (CPAP) ≥ 5 cm H_2_O
  **moderate:** PaO_2_/FIO_2 _= 100–199 mm Hg and PEEP ≥ 5 cm H_2_O
  **severe:** PaO_2_/FIO_2_ ≤ 100 mm Hg and PEEP ≥ 5 cm H_2_O.

All statements in the existing guideline were revised and the formulations were adapted pursuant to the Berlin definition.

## Preparation process

This guideline is the result of systematic literary research as well as the subsequent critical evaluation of evidence using scientific methods. The methodical approach of the guideline development process corresponds to the requirements for evidence-based medicine as they were defined by the AWMF as a standard. With respect to positioning, recently published papers were studied starting in 2005; the newly incorporated aspect of early mobilisation comprises all previously published literature up to and including 06/2014.

The guideline was prepared in the following steps:

1. Definition of the search terms for all topics of focus and determination of the relevant databases:
  **Pulmonary disorders:** (adult; acute) respiratory distress syndrome/ARDS, acute lung injury, severe lung injury, atelectasis, shock lung, acute respiratory failure, postoperative respiratory failure, lung failure, lung insufficiency, respiratory failure, respiratory insufficiency, ventilator-associated/induced lung injury, ventilator-associated/induced pneumonia, prevention/prophylaxis pneumonia.
  **Hospital infections:** cross infection, nosocomial infection, hospital infection.
  **Ventilated patients, intensive care patients**: critically ill, critical illness, catastrophic illness, critical care, intensive care, intensive care unit (ICU), respiratory care units, artificial respiration, mechanical ventilation.
  **Positioning:** prone position, supine position, lateral position, sitting/semi-seated position, horizontal position, semi-recumbent position, positioning, rotation, body position, patient positioning, positioning therapy, kinetic therapy, continuous lateral rotation, backrest elevation, axial/body position change, facedown position, side position, posture.
  **Early mobilisation**: early ambulation, accelerated ambulation, occupational therapy, physical therapy, mobility therapy, exercise therapy, early mobilisation, early exercise, early activity, physical therapy modalities.
2. Systematic research of scientific literature (University Library Regensburg), but also previously available guidelines, recommendations and expert opinions.
3. The evaluation of these publications according to the evidence criteria of the Oxford Centre for Evidence-based Medicine (levels of evidence, www.cebm.net, as of 2001). Due to the fact that the guideline is a revision and not a new development, this schema was also applied.
4. Consensus process

The first author of the guideline was employed as a speaker and commissioned by the DGAI committee to designate additional participants of the guideline group. In two consensus conferences as well as during two telephone conferences, the core statements and recommendations were coordinated with the entire guideline group under the direction of a moderator from AWMF by means of a nominal group process. The individual steps were recorded in entirety and editorially prepared by the speaker of the guideline group together with Dr. M. Bischoff and Ms. K. Gebhardt. The guideline was adopted by the DGAI committee on 30 April 2015.

### Members of the guideline group

The guideline was coordinated by the speaker of the group, Prof. Dr. Thomas Bein, Clinic for Anaesthesiology, University Hospital Regensburg.

Dr. Monika Nothacker, Association of Scientific Medical Societies (AWMF), Marburg assumed the methodological guidance of guideline development.

The guideline group comprised the following members:

Dr. Melanie Bischoff (DGAI), Uta Brückner (German Association for Physiotherapy), Kris Gebhardt (DGAI), Prof. Dr. Dietrich Henzler (DGAI), Carsten Hermes (German Association for Specialised Nursing Care and Functional Services), Prof. Dr. Klaus Lewandowski (DGAI), Prof. Dr. Martin Max (DGAI), Prof. Dr. Thomas Staudinger (Austrian Association for Internal and General Intensive Care Medicine and Emergency Medicine), Prof. Dr. Michael Tryba (DGAI), PD Dr. Steffen Weber-Carstens (DGAI) and Prof. Dr. Hermann Wrigge (DGAI).

### Selection of literature

Extensive literary research was conducted by the speaker of the guideline group at the University Library of Regensburg in collaboration with the director of the medical section (Dr. Helge Knüttel) based on preformulated keywords. The search was conducted via the German Institute for Medical Documentation and Information (DIMDI). This includes 40 extra databases in addition to Medline, Embase, Cochrane and SciSearch.

All papers published in the databases as of 17 May 2005 (final date of last research) were inspected. Only German or English-language publications were taken into account. The literary search primarily related to controlled studies, systematic reviews, meta-analyses, case series, case reports and comments/editorials. The focus was on publications involving adult patients. Articles from the paediatric field were only included if statements were recognised that enabled principle and age-independent statements. Only studies conducted on humans were included. Papers relating to animal experimentation were only evaluated if significant pathophysiological conclusions could be made regarding the functional principle of positioning therapy. Articles from textbooks were not used. Informational material from the medical device industry was only used for technical questions.

Initially, the literature of the already existing guideline was revised. Of the 287 publications included in the analysis at the time, only 170 articles were taken into consideration. A total of 117 articles (editorials, case reports and smaller studies) were precluded after updating the data if newer articles regarding the same subject matter had been published.

Within the scope of research (May 2005–May 2014), 7051 publications were initially identified based on the search terms. After viewing the abstracts, excluding duplicates and reviewing relevance, 952 publications were analysed at first. After reading the full texts, an additional 653 studies were precluded due to lacking relevance or inadequate study design (e.g. limited case numbers, probability for ‘bias’ statistical deficiencies) or lack of reference (experimental animal studies, paediatric patients). In the analysis, 299 studies were ultimately included and evaluated based on the aforementioned evidence schema. In the course of subsequently designating 29 relevant publications as well as a guideline (editorial deadline: 31 December 2014), ultimately 329 publications were analysed. Of these, 149 articles were included in the final version of the revision, which results in a total of 319 including the 170 articles adopted from the first version (Table 1).

**Table 1:** Characterisation of the literature used for the revision of the guideline

Overviews/reviews	47
Systematic reviews	25
Meta-analyses	16
Randomised controlled studies	32
Cohort studies/controlled case series	135
Editorials	10
Case reports	13
Experimental/animal experimental publications	6
Expert opinions	23
General overview	8
Guidelines/recommendations	4
**Total:**	319

### Organisational and methodological process of the preparation of the guideline

The preparation of the guideline was methodologically supported by Dr. Monika Nothacker, AWMF. In two conferences in June and November 2014 as well as during two telephone conferences in January and March 2015, the core statements of the existing guideline were revised by means of a nominal group process and recompiled with respect to early mobilisation. Roll call votes were not necessary with regard to the preparation of the S2e; there were no potential influencing factors due to interests linked to industrial products or other matters. Literary research and evaluation was prepared by the editorial team for the individual topics.

### Financing

Travel expenses within the scope consensus conferences and literary research were financed through the German Anaesthesiology Fund. Support was not provided from sponsors from the industry.

### Evidence level and recommendation grading schema

The classification of the Oxford Centre for Evidence-based Medicine (May 2001) was the basis for the evidence level and recommendation grading schema. It was modified and adapted for use in Germany [226] (see Tables 2 and 3)

**Table 2:** Evidence level schema

Source of evidence	Level
Methodologically suitable meta-analysis/analyses from RCTs	1a
Suitable RCT(s) with a small confidence interval	1b
Well-designed controlled trial(s) without randomisation	2a
Controlled cohort trial(s), RCT(s) of an unlimited method	2b
Uncontrolled cohort trial(s), case control trial(s)	3
Expert opinion(s), editorial(s), case reports(s)	4

*RCT* randomised controlled trial.

**Table 3:** Schema for grading recommendations

Evidence level	Recommendation classification	Recommendation grade
1a, 1b	Strong recommendation of ‘primary’ importance	A
2a, 2b	Moderate recommendation of ‘secondary’ importance	B
3, 4	Low recommendation, minimal clinical importance	0

### Explanation regarding recommendations

Recommendations are classified based on the best-available evidence and clinical assessment in a formal consensus process (nominal group process). Thus, the essential findings extracted from literature and assessed according to evidence are initially briefly outlined in the guidelines. The recommendation statement including the evaluation is then made. The grading of the recommendation is thus deducible and comprehensible from the previously presented and evaluated clinically scientific statements. Recommendation classifications may deviate from the evidence level if the guideline group deems this necessary based on ethical or clinical aspects, the evaluation of side effects or clinically practical application, for example in the case of cost/benefit considerations.

Furthermore, strong recommendations for therapeutic forms or methods may be expressed, for which the available evidence is not sufficient, but which are indispensable for the clinical process. On the other hand, methods or therapeutic principles, for which a strong recommendation would have to be expressed based on the studies, may receive a low recommendation grade due to their limited clinical importance. The reasons of such a deviating evaluation are mentioned in the text.

## Prone position in patients with acute pulmonary disorders

### Definition of prone position

The prone position implies the positioning of a patient by 180° from the supine position. An incomplete prone position means a position between approximately 135° and < 180°.

### Rational of the prone position

The primary goal of the prone position in patients with acute lung injury is to improve pulmonary gas exchange. Additional goals are to prevent/reduce the lung damage and secretion mobilisation. This involves a significant therapeutic method in addition to an optimised ventilation strategy [33, 62, 127, 163, 275] (evidence level 1a).

### Physiological fundamentals: effects of the prone position

The significant physiological effects of the prone position are: (a) changes of the respiratory mechanics, (b) the reduction of the pleural pressure gradient [126, 127, 166, 183, 206, 227] and (c) the reduction of tidal hyperinflation [62] as well as the ventilation induced lung injury (‘stress and strain’) [193]. They may lead to the homogenisation of pulmonary gas exchange [5, 102, 203], to a reduction of ventilation-perfusion mismatch [102, 215], to an increase of lung volume involved in gas exchange in CT analyses due to a reduction of marginally or non-ventilated areas (atelectasis) [104, 107] and to a reduction of ventilation-associated lung injury [5, 45, 46, 194, 228, 278]. The assumption is made that an improvement of the drainage of bronchoalveolar secretion is affected.

Regarding (a): In ventilated patients with acute lung failure, the prone position leads to a reduction of thoracoabdominal compliance [227, 282]. Repositioning to the supine position leads to a general increase in compliance of the entire respiratory system compared to the previous supine or prone position [227, 261]. This effect becomes more distinctive the higher the elastance of the thorax and diaphragm (thoracoabdominal compliance) is at the beginning of the positioning method (evidence level 2a).

Regarding (b): The prone position leads to a homogenisation of pulmonary gas dispersion in healthy lungs [215] as well as in the case of acute respiratory insufficiency [102, 124, 204, 298] and pulmonary perfusion [147, 216, 252] and thus improves the overall ventilation/perfusion ratio [166, 203, 216, 225] (evidence level 2b). In some ventilated patients with an acute limitation of the pulmonary gas exchange, the prone position may cause an increase of gas exchanging lung tissue (recruitment) through a reduction of atelectatic areas of the lungs. The significance of this effect overall is still unclear [6, 62, 104, 123] (evidence level 2b).

Regarding (c): Ventilation in the prone position leads to a delay and reduction of ventilation-induced lung injury in animal experimentation [45, 46, 293] as well as in patients with acute lung damage [62, 193] compared to ventilation in the supine position (evidence level 2b). It is assumed that an increase of drainage of bronchoalveolar secretion is caused by the prone position, however there is no data to support this hypothesis (evidence level 4).

### Effects of the prone position on the pulmonary gas exchange

In patients with acute respiratory insufficiency and particularly in the stage of ARDS, ventilation in the prone position leads to an acute increase of arterial oxygenation if the settings of the ventilation device are not changed [1, 2, 8, 33, 36, 54, 91, 96, 100, 105, 123, 125, 146, 167, 171, 175, 177, 185, 204, 225, 245, 261, 266, 271, 273–275, 280, 288, 302, 307] (evidence level 1a). Not all patients experience an acute improvement of oxygenation in the prone position; the rate of nonresponsiveness (absence of an increase in oxygenation by > 20 % of the initial value for several hours after situation in the prone position) is not systematically studied. The underlying disease, the time of onset and the type of application (length of time in prone position, positioning intervals) are of great significance for the effect (see below) [297]. Some patients experience increased CO_2_ elimination during ventilation in the prone position if the settings of the ventilation device remain unchanged, possibly as an expression of a recruitment [106, 124, 236] (evidence level 3).

### Effect of the prone position on the duration of ventilation, incidence of pneumonia, length of hospitalisation and mortality

In two broad studies from multiple centres, daily prone positioning (approximately 8 h for 5–10 days) did not lead to a significantly shorter ventilation period or to a survival advantage in patients with modest to moderate ARDS (PaO_2_/FIO_2 _< 300 mm Hg) despite an increase of oxygenation compared to patients who were not placed in the prone position [105, 126] (evidence level 2b). Likewise, until then this did not reveal a shorter duration in intensive care or hospital treatment. In the most severe case of ARDS (PaO_2_/FIO_2 _< 88 mm Hg), however, a post-hoc analysis [105] revealed a survival advantage through daily prone positioning compared to patients, who were not placed in the prone position (evidence level 2b). In one study, the occurrence of ventilator-associated pneumonia (VAP) was substantially lower in patients, who were repeatedly placed in the prone position [124]. In one prospective observational study [200], no reduction of VAP incidence could be demonstrated (evidence level 3).

In more recent studies conducted by multiple centres, patients with ARDS in an early stage of the disease spent approximately 20 h a day in the prone position. A trend appeared involving a shorter period of ventilation and a higher rate of survival (evidence level 2b), however, the studies revealed design flaws or a heterogeneous patient group [91, 185, 280]. These studies were compiled and interpreted in meta-analyses; an overview of the meta-analyses from 2008–2014 can be found in Table 4.

**Table 4:** Meta-analyses (2008–2014) regarding randomised trials ‘prone position in ARDS patients’. The specification ‘ml/kg’ refers to ‘ideal body weight’ (‘predicted body weight’)

	Design/Goal	Patients	Result
Alsaghir and Martin [8]	Mortality, PaO_2_/FIO_2,_ Duration of ventilation, VAP incidence	5 studies: 1316 patients	No effect on Mortality Sub-analysis: SAPS-II 50: mortality ↓ PaO_2_/FIO_2_ ↑ No effect on the duration of ventilation or VAP incidence
Sud et al. [274]	ICU + 28-day mortality, PaO_2_/FIO_2_, duration of ventilation, VAP, complications	13 studies: 1559 patients	No effect on mortality PaO_2_/FIO_2_ ↑ No effect on VAP
Abroug et al. [2]	28-day mortality, PaO_2_/FIO_2,_ VAP incidence, ICU duration, Complications	6 studies: 1372 patients	Broad variation in study design No effect on mortality PaO_2_/FIO_2_ ↑ No increased complication rate No significant VAP reduction
Kopterides et al. [163]	Mortality, duration of ventilation, complications	4 studies: 1271 patients	No effect on mortality Increased complication rate in the prone position
Sud et al. [273]	Hospital mortality: PaO_2_/FIO_2_ 100 versus PaO_2_/FIO_2_ ≤ 100 (prone position at the onset) versus supine position	10 studies: 1867 patients	Hospital mortality significantly reduced in patients with PaO_2_/FIO_2_ < 100 prone position at the onset
Abroug et al. [1]	ICU and hospital mortality, complications	7 studies: 1675 patients	Inhomogeneity of patients and study design No effect on overall mortality Reduction of ICU mortality in 4 studies No increased complication rate
Beitler et al. [33]	60-day mortality with stratification: Tidal volume 8 ml/kg versus ≤ 8 ml/kg	7 studies with 2119 patients	No reduction of mortality for the entire group, but a significant reduction for the ‘low tidal volume’ group (≤ 8 ml/kg)
Sud et al. [272]	Mortality in patients in the prone position and lung protective ventilation	11 studies: 2341 patients. Including 6 studies: 1016 patients ventilated for the protection of the lungs	Significant reduction of mortality through the prone position in patients with a lung protective ventilation strategy

*ARDS* acute respiratory distress syndrome, *VAP* ventilator-associated pneumonia, *ICU* intensive care unit.

In one multicentre study with a prospective randomised design [127], 237 patients with moderate or severe ARDS were placed in the position soon (< 48 h) following the occurrence of the disease (16 h or more daily for approximately 7 days), while the patients from the control group were treated in the supine position. All patients were ventilated lung protective and received muscle relaxants at an early stage of the ARDS. Ninety-day mortality was 23.6 % in the group of those in the prone position and 41 % in the control group (*p* < 0.001, Odds Ratio (OR) = 0.44). The occurrence of complications did not differ between the groups, although the control group patients demonstrated a substantially higher occurrence of cardiac arrhythmias (evidence level 1a).

**In patients with ARDS (PaO**_**2**_**/FIO**_**2**_< **150) and a lung-protective ventilation strategy, the early application of a prolonged prone position leads to a substantial decrease in mortality compared to the supine position (evidence level 1a). It is not clear, whether or not repeated prone positioning is suitable for decreasing the incidence of nosocomial pneumonia (evidence level 4).**

**►1 Patients with ARDS and an impairment of arterial oxygenation (PaO**_**2**_**/FIO**_**2**_< **150) should be placed in the prone position (evidence level 1a, recommendation grade A).**

### Time and duration of the prone position

The positive effect of the prone position on the gas exchange may occur immediately (≤ 30 min) or with a delay of up to 24 h after repositioning [36, 100, 169, 188, 242] (evidence level 2b). A shorter anamnesis of the ARDS was associated with a more positive effect of the prone position on oxygenation and outcome [125, 126] (evidence level 1b). The extent of initial improvement of oxygenation does not permit a prognosis for a ‘long-term effect’ (e.g. after 12 h) [242]. Likewise, there is no typical morphology in thoracic CT for the prognosis of success in the prone position [221] (evidence level 3b).

Multiple intervals of an intermittent prone position and supine position revealed a sustainable effect for the improvement of the pulmonary gas exchange (in the supine position) compared to a method conducted once [100, 105, 125] (evidence level 2b). In comparison to continuous axial rotation, treating ARDS patients with prone positioning leads to a more rapid and distinctive increase of oxygenation, although a difference between the patient groups is no longer demonstrable after 72 h [266] (evidence level 2b).

**►2 A prone positioning interval of at least 16 h should be targeted. The prone position should be considered at an early stage and implemented immediately after indication (evidence level 2b, recommendation grade B).**

**►3 Prone positioning should be concluded in the case of persistent improvement of oxygenation in the supine position (4 h after supine positioning: PaO**_**2**_**/FIO**_**2**_ **≥ 150 with a PEEP ≤ 10 cm H**_**2**_**O and FIO**_**2**_ **≤ 0.6) or if multiple positioning attempts remained unsuccessful (evidence level 3, recommendation grade B).**

### Synergy effects of the prone position with additional measures

The improvement of oxygenation in the prone position is reinforced through the application of PEEP, particularly in the case of diffuse ARDS [62, 101] (evidence level 2b). Intermittent recruitment manoeuvres lead to a more sustainable effect on oxygenation while in the prone position as opposed to the supine position [102, 227] (evidence level 2b). The integration of spontaneous respiratory rates while in the prone position, for example through the application of biphasic positive pressure ventilation with spontaneous respiration (‘airway pressure release ventilation’ [APRV]), increased the effect of positioning methods compared to ventilation in a predominantly controlled mode [295] (evidence level 2b). The inhalation of nitric oxide for the improvement of the ventilations/perfusion ratio [39, 111, 114, 145, 186, 220, 243] likewise demonstrated synergetic effects on oxygenation (evidence level 2b).

Ventilation in the prone position presents a sensible therapeutic perspective in order to implement a lung-protective strategy by adapting various ventilation settings parameters (reduction of the tidal volume, reduction of FIO_2_, the inspiratory peak pressure, as well as the pressure difference in inspiration and expiration). Moreover, ventilation in the prone position implies physiological protection/reduction of ventilation-associated lung injury [102, 107, 124, 127, 170, 193] (evidence level 2b).

**►4 The same principles of an optimised ventilation strategy apply for ventilation in the prone position as for the supine position, including the lung-protective limitation of tidal volume, the prevention of derecruitment and the integration of spontaneous respiratory rates (evidence level 2b, recommendation grade A).**

**►5 An evaluation and adjustment of the ventilation mode in the context of a lung-protective strategy should be conducted after each change of position (evidence level 3, recommendation grade B).**

### Effect of the prone position on other organ systems

Prone positioning per se is not a method that promotes hypotension or cardiac instability [134, 146, 149, 193, 299] (evidence level 1b). In a broad study, prone positioning—as opposed to supine positioning—lead to an improvement of haemodynamics (increase of cardiac output or median arterial pressure) and to a reduction of cardiovascular complications [125], however, a balanced volume status was necessary for this effect [149] (evidence level 2b). In patients without a pre-existing limitation of the renal function, prone positioning did not lead to a reduction of kidney function [134] (evidence level 2b). Positioning on mattress systems controlled by compressed air reduced a positioning-related increase of intra-abdominal pressure compared to conventional mattress systems [58, 198] (evidence level 2b). Patients with abdominal obesity (CT definition: sagittal abdominal diameter ≥ 26 cm) developed kidney failure (83 vs 35 %, *p* < 0.01) [309] at a significantly higher rate during prolonged prone positioning (on average 40 h) compared to patients without a similar configuration (evidence level 2b).

**►6 Prior to the application of prone positioning, the patient should be stabilised haemodynamically and the volume status should be balanced. The use of catecholamines is not a contraindication against the prone position (evidence level 2b, recommendation grade B).**

In patients demonstrating no abdominal disease, a minimal, though substantial increase of intra-abdominal pressure without intra-abdominal compartment syndrome occurred as a result of prone positioning during a period of up to 2 h [99, 134, 135] (evidence level 2b). Likewise, no impact on splanchnic perfusion was demonstrated [157, 187]. There are no study results for patients with acute abdominal diseases and increase of pressure. There have been just as few previous reports that the type of abdominal positioning (padded vs hanging) or the duration of positioning has an influence on intra-abdominal pressure or perfusion ratios [58, 61, 134, 205], although this type of support of the thorax and pelvis worsened the compliance of the thoracic wall and increased pleural pressure (evidence level 2b). Patients with abdominal obesity developed hypoxic hepatitis during prolonged periods in the prone position (on average 40 h) at a significantly higher rate than patients without a similar configuration (22 vs 2 %, *p* = 0.015) [309] (evidence level 2b).

**For patients with acute abdominal diseases, no recommendation can currently be provided with respect to the type and duration of a prone position due to the lack of studies (evidence level 4, recommendation grade 0)**

**►7 CAVE: In patients with abdominal obesity, kidney and liver function should be monitored closely in the event of prolonged prone positioning (expert consensus).**

### Prone positioning and acute cerebral lesion

Prone positioning may cause an increase of intracranial pressure and (in the case of unchanged haemodynamics) a reduction of cerebral perfusion pressure in the case of acute traumatic or non-traumatic cerebral lesions [34, 209, 241] (evidence level 4). However, the improvement of the pulmonary gas exchange induced by the prone position may increase cerebral oxygenation [283] (evidence level 4). In healthy humans, systematic and cerebral haemodynamics were captured in the prone position during noninvasive positive pressure ventilation and a variation of the position of the head was conducted (centred, to the left and right side). The lateral rotation of the head leads to a reduction of cerebral blood flow (Arteria cerebri media) by approximately 10 % [137] (evidence level 2b).

Sufficient studies were not conducted previously as to whether or not an adaption of the ventilation settings (change of tidal volume and respiratory minute volume = change of CO_2_ elimination = change of cerebral perfusion) could have positive effects on the damaged cerebrum while in the prone position. Moreover, no study has been conducted regarding whether or not the adapted analgosedation could prevent the intracranial pressure increase in the case of an acute cerebral lesion.

**►8 The indication for the prone position with acute cerebral lesions may only be issued after individual consideration of benefit (improvement of oxygenation) and risk (intracranial pressure increase) (evidence level 3, recommendation grade 0).**

**►9 During the positioning method, intracranial pressure should be continuously monitored (evidence level 2b, recommendation grade A). The head should be centred during this method and lateral rotation should be avoided (evidence level 3, recommendation grade B). Expert consensus and S1 guideline***Intracranial Pressure***(AWMF registry no. 030/105, valid until 12/2015).**

### Prone positioning and intraocular pressure

In one prospective, randomised trial, intraocular pressure (IOP) was measured in patients in the prone position in an operative area prior to, during and after the positioning method, wherein the heads of a patient group were additionally turned to the right side at a 45° angle to the prone position [73]. While in the prone position, a moderate increase of the IOP occurred from 12 to 18 mm Hg (*p* < 0.001) and upon turning the head to the side, the pressure of the lower eye increased further. Two additional studies from the operative area confirmed these findings [88, 122] (evidence level 2b). There is no data in this regard for intensive care patients.

### Modifications of the prone position

In addition to the complete prone position (180^°^), the ‘incomplete’ prone position (135^°^) is also applied because it is perceived as having fewer side effects for patients and is easier to perform for the nursing staff [30, 257]. With proper execution, there were no significant differences between both positions in the incidence of severe complications [30] (evidence level 2b).

The incomplete prone position lead to a substantial improvement of oxygenation in ARDS patients; however, this effect was not as distinctive as with the complete prone position. In patients with severe ARDS, a significant increase of arterial oxygenation (defined as an improvement by more than 20 %) while in a complete prone position occurred at a significantly higher rate than while in the 135^°^ prone position [30] (evidence level 2b). In one prospective randomised study, the combination of the prone position with an elevation of the upper body lead to a significantly stronger effect on the oxygenation compared to the prone position alone [245] (evidence level 3).

**►10 The complete prone position has a stronger effect on the oxygenation than the incomplete prone position and should be primarily applied (evidence level 2b, recommendation grade A).**

**►11 The elevation of the upper body while in the prone position may be sensible for preventing an impact on other organs (intraocular pressure, intracranial pressure) (evidence level 3, recommendation grade 0).**

### Complications while in the prone position

The following complications were described while in the prone positions [28, 30, 42, 43, 65, 105, 124, 144, 218, 272, 301] facial oedema (20–30 %), pressure ulcers around the face/cornea, pelvis, knee (approximately 20 %) [234] ‘intolerance’ while in the prone position (= coughing, compaction, respiratory problems approximately 20 %), cardiac dysrhythmias (approximately 5 %), necrosis of the mamilla, pressure ulcers of the tibial crest (individual reports), dislocations of the tracheal tube or venous/arterial lines (approximately 1–2 %) [105], nerve damage (two case studies regarding brachial plexus lesion [119]) (evidence level 2b). In this regard, it is necessary to consider that complications also occur in the supine position and a comparison of the incidences of position-related complications for the prone position has not previously been sufficiently studied. The retrospective analysis of the multicentre study by Guerin [116] revealed a higher incidence of pressure points and skin ulcers in the prone position group (14.3/1000 ventilation days) compared to the supine position (7.7/1000 ventilation days, *p* = 0.002) (evidence level 2b).

According to the results of a prospective, randomised study, a lesser frequency of facial oedema was observed due to the modification of the prone position (135^°^ position, ‘incomplete prone position’) compared to the 180^°^ position [30] (evidence level 2b). The safe execution of the prone position in patients with extracorporeal membrane oxygenation (ECMO) was reported in a retrospective observational study [158] (evidence level 3).

### Contraindications for prone positioning

Instability of the spine, severe, surgically untreated facial trauma, the acute cerebral lesion with intracranial pressure increase, the critical cardiac rhythm disorder, acute shock syndrome and the ‘open abdomen’ situation apply as contraindications for prone positioning [304, 306].

**►12 Compared to the supine position, the prone position leads to a higher incidence of pressure ulcers and respiratory problems, such that a positioning should be done particularly gentle and the airways should be protected and monitored (evidence level 2, recommendation grade A).**

**►13 An open abdomen, spinal instability, increased intracranial pressure, critical cardiac rhythm disorders and manifest shock are contraindications for the prone position. These contraindications may be deviated from in individual cases after consideration for the benefits and risks and following consultation with the specialist disciplines involved (expert consensus, recommendation grade 0).**

### Appendix I: Prone positioning: recommendations for practical execution

#### Prone positioning: practical execution

Each positioning process—depending on the body weight of the patient as well as the invasiveness of the therapy (drainages, catheters, extensions)—is conducted by three to five nurses and one physician [13, 17, 18, 42, 138, 190, 195, 207, 254, 260, 276, 303, 304].

A. Preparational measures:
  1. Within the scope of prone positioning, the use of a special anti-decubitus mattress system is recommended to prevent/reduce pressure ulcers (evidence level 4, recommendation grade 0), particularly in patients with an increased decubitus risk (high-dose catecholamine therapy, adiposity, cachexia, corticosteroid therapy) (evidence level 3, recommendation grade 0).
  2. Catheters, drainages and artificial airways are secured and, if necessary, extended. Prior to positioning, it is necessary to check whether or not it is a ‘difficult-airway-situation’ in order to take potentially suitable measures to ensure the airways (e.g. preventative surgical tracheotomy, providing intubation alternatives). When performing the rotation, the most essential access points should be secured by the person guiding the head of the patient.
  3. The inspiratory fractional oxygen concentration (FIO_2_) should be set to 1.0.
  4. Enteral nutrition is interrupted; the stomach should be emptied through a tube.
  5. An adapted analgosedation (Richmond Agitation Sedation Scale (RASS-Score) ≤ − 2) is necessary for the rotational manoeuvre to avoid coughing, compaction or regurgitation. Ventilation should be customised accordingly. After the positioning manoeuvre, the analgosedation is reduced.
B. Execution

During the rotating manoeuvre, monitoring is necessary by means of continuous arterial blood pressure measurement. Various techniques are described for executing the rotating process. It is recommended to focus on one technique that all involved are familiar with [13, 195] (evidence level 4, recommendation grade B for all previously described methods).

C. Follow-up
  1. After the completed positioning manoeuvre, monitoring must be completed.
  2. Ventilation must be adapted in the context of a lung-protective strategy and monitored after a brief stabilisation phase (evidence level 3, recommendation grade B).
  3. After the rotating manoeuvre, special measures are taken to reduce pressure around the head, around the pelvis and the knee. Always ensure careful padding particularly in areas prone to decubitus (recommendation grade A). The head and arms should be additionally repositioned in short intervals while in the prone position (recommendation grade 0).
D. Special aspects for executing prone positioning:
  1. The application of enteral nutrition while in the prone position was studies in multiple trials [240, 255, 294]. In one prospective trial, the residual gastric volume while in the prone position was greater than in the supine position [240]. In another trial, with adequate enteral feeding tube length, no increased residual gastric volume or an increased incidence of regurgitation was observed in contrast to the supine position [255] (evidence level 2b). On the condition of an application with a low flow rate (≤ 30 ml/h) and frequent reflux checks, no higher residual volumes or other side effects were observed in one prospective trial [294] (evidence level 2b), this approach is recommended in a systematic analysis [178].
  2. While in the prone position, enteral nutrition is possible with a low flow rate (≤ 30 ml/h), however regular reflux checks are suggested (evidence level 2b, recommendation grade B).

## Continuous lateral rotation therapy

### Definition of continual lateral rotation therapy (CLRT)

CLRT involves the continuous rotation of the patient around his longitudinal axis in a motor-driven bed system. Depending on the system, a maximum rotational angle of 62° can be achieved on each side.

### Rational of CLRT

The goals of CLRT are to prevent pulmonary complications (atelectasis, pneumonia, congestion of pulmonary secretion), the reduction of pulmonary inflammation as a result of trauma or infection, as well as improving pulmonary gas exchange in ventilated patients. The increase of oxygenation, the incidence of nosocomial pneumonia, as well as the duration of mechanical ventilation and intensive care stays or hospitalisation are classified as parameters for this. However, none of these parameters are established as an adequate surrogate for survival and the quality of survival. Indications for the use of CLRT comprise both prophylactic (prevention of complications) and therapeutic aspects (improvement of pulmonary functionality).

*Comment: In one recommendation from the Paul Ehrlich Society (PEG) ‘Nosocomial Pneumonia: Prevention, Diagnostics, und Therapy’* [38]*, there is no recommendation for the use of CLRT within the scope of a ‘bundle’ for the prevention of ventilator-associated pneumonia. The current recommendations of the Commission for Hospital Hygiene and Infection Prevention (KRINKO) at the Robert Koch Institute* [162] *determined based on ‘lacking consistency’ in the trials and meta-analyses that, ‘Therapy with kinetic beds for the prevention of VAP (“ventilator- associated pneumonia”) cannot be recommended at this time.’ As a restriction to this recommendation, it is necessary to adhere to the fact that at the time of the publication from the KRINKO, the prospective randomised publications from Staudinger et al.* [265*] and Simonis et al.* [263] *were not yet published.*

The use of CLRT requires a targeted indication and safe handling in order to prevent undesired effects. After initiating this method, the persistence of the indication—as with other therapeutic methods as well—should be reviewed daily.

### Effects of CLRT on pneumonia incidence, duration of ventilation and mortality

The present studies regarding the effect of CLRT on the incidence of respiratory infections are limited by various criteria for the diagnosis of infections of the upper and lower respiratory tracts as well as the lung parenchyma [70, 71, 120, 136, 184, 263, 265].

In two more recent prospective randomised trials [263, 265], a reduction of the incidence of respiratory infection including ‘ventilator-associated pneumonia’ (VAP) was observed in ventilated patients compared to standard positioning (bedsore prophylaxis) (evidence level 1b). Furthermore, in the study by Staudinger et al. [265], the ventilation time (8 vs 13 days, *p* = 0.02) and the treatment time in intensive care (25 vs 39 days, *p* = 0.01) was significantly shorter in patients treated with CLRT; the mortality rate did not differ. The study by Simonis et al. on patients in cardiogenic shock [263] demonstrated—in addition to VAP reduction—a significantly higher 1-year survival rate (59 %) compared to the control group without CLRT (34 %, *p* = 0.028) (evidence level 1b). There are no comparative studies of CLRT with other positioning methods for preventing VAP.

**►14 The early use of CLRT can be employed in certain groups of ventilated patients as a supplement to prevention of ventilator-associated pneumonia, however, other methods (e.g. adapted analgosedation, mobilisation concepts) should not be impacted by this (evidence level 3, recommendation grade B).**

The treatment period in intensive care was shorter in three out of eight randomised trials compared to conventionally treated patients (evidence level 1b). The length of hospitalisation was shortened due to CLRT in a prospective randomised trial [265] (evidence level 1a), though not in other trials with partially limited quality [4, 60, 211, 291] (evidence level 3).

### Physiological effects of CLRT

CLRT was originally used in immobilised patients for bedsore prophylaxis. Subsequently, the indication was broadened for the treatment of patients with pulmonary disorders. Improved oxygenation, the dissolution of atelectasis, improved ventilation/perfusion ratios, increased secretion mobilisation, the reduction of pulmonary inflammatory response following trauma and a reduction of pulmonary fluid retention was determined as effects.

### Effects of CLRT on the pulmonary function

CLRT improves the pulmonary gas exchange in patients with acute respiratory insufficiency (evidence level 2b) [25, 222, 223, 237, 238, 265, 267]. The following effects were confirmed starting at a rotational angle of ≥ 40° on each side:

- The reduction of extravascular lung water (EVLW) in patients with impaired oxygenation (ARDS) [32] (evidence level 2b). The mechanism is not ultimately clear; continual movement and changes in intrapulmonary pressure ratios possibly lead to increased drainage through the lymphatic system of the lungs [10, 29] (evidence level 4).
- The reduction of ventilation/perfusion mismatch [27] (evidence level 4).
- In some trials, the incidence and extent of atelectasis were reduced with the early, that is preventative use of CLRT from the start of ventilation. Few limitations of oxygenation occurred [4, 98, 160]. In other trials, however, no significant effects were demonstrated [51, 110, 277, 310] (evidence level 3). Particularly in polytraumatised patients with a pulmonary injury, early CLRT was able to prevent the occurrence of ARDS or improve oxygenation [31, 86, 93, 202, 223, 300] (evidence level 2b).
- In trauma patients, CLRT reduced the pulmonary inflammation reaction (reduction of pulmonary and systemic pro-inflammatory cytokines (TNF, IL-6) and lead to a less severe organ function disorder up to the fifth day post-trauma compared to patients treated in the supine position [31] (evidence level 2b).
- In one trial, CLRT lead to the dissolution of atelectasis in ventilated patients [238]; a more recent publication could not verify this effect [51], however both studies demonstrate methodological weaknesses. Thus, no recommendation is provided for treating atelectasis with CLRT.
- The improvement of oxygenation due to CLRT in patients with restricted respiratory function (ARDS) occurred at a slower rate than in the prone position [266] (evidence level 2b).
- To date, there has been no proof of increased bronchopulmonary secretolysis due to CLRT; however, a rotational angle of < 30° was used in the only study [77] (evidence level 4).

**►15 CLRT should not be used in patients with ARDS (PaO**_**2**_**/FIO**_**2**_ < **150) (recommendation grade A).**

**In the case of contraindications to the prone position, the use of CLRT may be considered for improving oxygenation (evidence level 3, recommendation grade 0).**

### Time and duration of CLRT: angular settings

In most studies, CLRT was conducted at beginning of intensive care treatment for at least 72 h. The use of CLRT within 2 days after development of a respiratory insufficiency was linked to a significant reduction of intensive care therapy and hospitalisation compared to a later initiation of the method in two studies [98, 279] (evidence level 3). One positive effect on the gas exchange was able to be observed up to a duration of 5 days after the onset of treatment [25, 224] (evidence level 4). The parameters or strategies according to which CLRT should be concluded have not been studied (‘Weaning’) [94].

In one study, it was determined that longer periods of retention in the lateral position during CLRT do not improve the gas exchange and may even cause a deterioration in individual cases due to a reduction of pulmonary compliance [256 (evidence level 2b). The positive effects on oxygenation and on pneumonia incidence (see below) were observed with one exception [310] during CLRT with a rotational angle > 40°.

**►16 If CLRT is used for treating oxygenation impairment, the indication for continuation should be reviewed daily based on the improvement of oxygenation (as with the prone position).**

**CLRT should be concluded upon stabilisation of the gas exchange in the supine position without rotation, or if a continuous application showed no success over a period of 48 h to no more than 72 h (evidence level 3, recommendation grade B).**

### Ventilation setting during CLRT and duration of CLRT

**►17 For ventilation during CLRT, the principles of a lung-protective ventilation strategy should apply (evidence level 2b, recommendation grade A).**

### Complications and interactions of CLRT

The following complications were described during CLRT: pressure ulcers, ‘intolerance’ (coughing, compactions, respiratory problems), cinetosis, catheter dislocations, nerve damage [93, 184, 277]. In one prospective observational trial on 20 ‘haemodynamically stable’ patients, no changes of heart rate or blood pressure were registered during CLRT [12] (evidence level 3). In the case of haemodynamically unstable patients, a drop in blood pressure in a steep lateral position (most often in the right lateral position) is frequently observed [26] (evidence level 2b). A direct comparison of the incidence of position-related complications with other positioning methods is not possible due to a lack of data.

There is data from two trials regarding the use of CLRT in patients with acute cerebral lesions [60, 287]. No increase of intracranial pressure during CLRT was stated in one trial [287] (evidence level 4).

In one retrospective trial, an increased complication rate and duration of ventilation during CLRT was determined in patients with spinal lesions, however the severity of neurological deficits in these patients was greater [57] than in the ‘conventionally’ treated group (evidence level 4).

**►18 The same criteria as with the prone position apply for conducting CLRT in patients with acute cerebral lesions. These patients should be monitored by means of continuous intracranial pressure measurement (evidence level 3b, recommendation grade 0) and may be situated in a moderately high upper body position (inclined position of the bed system).**

**►19 It is necessary to individually consider between potential damage due to CLRT and the expected benefit in the case of severely injured patients (evidence level 4, recommendation grade 0).**

### Contraindications for CLRT

An instable spine, acute shock syndrome and a body weight > 159 kg (according to the manufacturer) are considered to be contraindications for CLRT.

### Appendix II: continuous lateral rotation therapy: recommendations for practical execution

Careful positioning requires special protective measures for pressure-sensitive areas (head/neck, auricles, pelvis, knee, brachial nerve, peroneal nerve) [94, 224] (evidence level 4, recommendation grade B).

Prior to starting the system each time, a manual ‘test rotation’ should be conducted to check the proper positioning of the patient as well as adequate extension and attachment of all supply lines and drainages. CLRT should be started with small rotational angles and then increased. To achieve optimal rotational periods (18–20 h/day), nursing and physician activities should be well coordinated with each other (evidence level 4, recommendation grade 0). In the case of an invasive, continuous blood pressure measurement, the pressure sensor must be fastened to the bed system at the level of the heart in the median axis in order prevent false measurements during the rotational process. With a proper routine and preparation, CLRT can also be safely used in combination with extracorporeal membrane oxygenation [164] (evidence level 3, recommendation grade 0). In the case of distinctive haemodynamic insufficiency in the lateral position, the angle of rotation should be reduced to the respective side (recommendation grade 0).

## Lateral position for patients with pulmonary disorders

### Definition of lateral position

A position, in which the side of the body is supported and elevated up to an angle of 90°, is referred to a lateral position.

### Rational of the lateral position

In addition to relieving support areas (decubitus prophylaxis), pulmonary complications are intended to be prevented and the pulmonary gas exchange improved. This is the result of frequent repositioning or special lateral positioning in the case of unilateral lung damage. The simplicity of the method is beneficial, which can be conducted at any time with minimal additional effort [14, 141].

### Physiological effects and side effects of the lateral position in patients without lung damage

Effects on haemodynamics and gas exchange were studied, wherein primarily postoperative patients with healthy lungs were studied [50, 212].

Only minimal changes in ventilation and haemodynamics were detected in spontaneous respiration among individuals with healthy lungs [50]. Blood pressure tend to sink in the lateral position (left lateral position >right lateral position [148], evidence level 4). In the left lateral position, greater heterogeneity of ventilation dispersion occurred compared to the right lateral position [92] (evidence level 4). The lateral position promoted the perfusion in the direction of the ventral pulmonary sections in ventilated patients [27] (evidence level 3). The measurement of haemodynamics in the lateral position was vulnerable to artefacts, particularly when determining the reference point [12, 49] (evidence level 4).

In postoperatively ventilated patients without acute respiratory insufficiency, the overall compliance of the respiratory system in the lateral position is reduced compared to the supine position [282] (evidence level 4). The phenomenon of atelectasis formation after the induction of anaesthesia and atelectasis treatment through PEEP occurred in the dependent lung in the lateral position just as in the supine position [161] (evidence level 4).

In postoperatively ventilated patients with healthy lungs and without acute respiratory insufficiency, without atelectasis and with a high tidal volume, the lateral position (45^°^–90^°^) did not improve the pulmonary gas exchange compared to the supine position [212, 285, 286] (evidence level 2b). The moderate lateral position (45°) did not affect any clinical changes of the gas exchange, haemodynamics and tissue perfusion compared to the supine position [21, 285, 286] (evidence level 4). The mixed venous oxygen saturation decreased minimally [108] (evidence level 4).

The haemodynamics are only slightly influenced by the lateral position of ventilated patients; no significant changes of cardiac output occurred [22, 285, 286] (evidence level 4). A prophylactic effect of the lateral position on the prevention of postoperative pulmonary complications was not adequately studied.

**►20 During the ventilation of patients without lung damage, a lateral position exclusively for preventing pulmonary complications is not sensible (evidence level 2b, recommendation grade B).**

### Indications and effects of the lateral position in patients with lung damage

#### Bilateral lung damage

In the case of chronic obstructive pulmonary disease (COPD), noninvasive ventilation in the lateral position is possible. However, it does not cause any additional improvement of the gas exchange compared to the supine position [233] (evidence level 4). In two trials involving a total of 22 ventilated patients with acute lung damage, the effects on oxygenation due to the lateral position were variable and not predictable compared to the supine position [210, 256] (evidence level 4).

CLRT with a minimal rotational angle ≤ 40° and the intermittent, 2 h long lateral position had the same effect on the gas exchange, wherein higher secretion mobilisation was observed using CLRT [68] (evidence level 2b). In the right lateral position, there was more often a haemodynamic compromise in ventilated patients compared to the left lateral position caused by a more reduced right ventricular filling [26, 76, 120] (evidence level 2b). These effects have not been studied in non-ventilated patients or ventilated patients without lung damage.

**The effects of an intermittent lateral position or CLRT up to a rotational angle** < **40° on the pulmonary gas exchange have not been adequately verified. In patients with ARDS, CLRT up to 40° does not demonstrate any advantage compared to intermittent lateral positioning with respect to improving oxygenation (evidence level 2b).**

**►21 Proper positioning and interpretation of invasively measured blood pressure values should be particularly ensured in the lateral position (evidence level 3, recommendation grade B).**

#### Unilateral lung damage

In spontaneous breathing, the lateral position improves oxygenation if the good lung is down [23, 95, 284] (evidence level 4). However, in the case of a very high ‘closing volume’ it may be better to position the bad lung down [59] (evidence level 2b). Effects can be expected particularly with pneumonia, although not with central obstructions, such as carcinoma [53] (evidence level 4).

In the case of mechanical ventilation and lateral positioning with the good lung down, oxygenation improves [53, 59, 79, 143, 235, 244] (evidence level 2b) through homogenisation of ventilation/perfusion dispersion and reduction of the intrapulmonary shunt [115, 132] (evidence level 4). These improvements of the gas exchange are based on the same mechanisms as with the prone position, with which the bad lung is taken from the dependent position. These effects can be expected for gas exchange disorders due to pneumonia and atelectasis, but not due to pleural effusion [50] (evidence level 4). Effects of the lateral position on the outcome with respect to ventilation duration, pneumonia incidence or mortality have not been studied.

**►22 In the case of ventilation of patients with unilateral lung damage, a lateral position of approximately 90° is recommended with the good lung down to improve the gas exchange (evidence level 2b, recommendation grade B)**

## Backrest elevation position

### Definitions of elevated upper body position

**Fig. 1:**
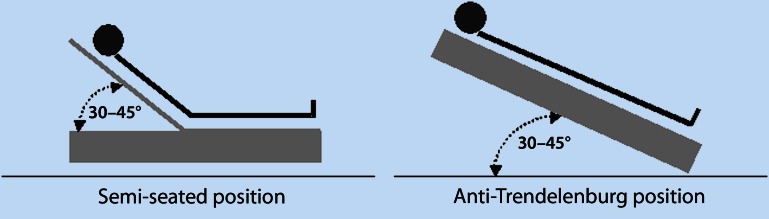
Modifications of the elevated upper body position

The elevated upper body position is implemented in various ways in different trials—there is no universal definition. Various positions are studied, which can range between the classic sitting position with bent hip and knee joints on one hand and tilting of the entire, flat-lying patient (called the anti-Trendelenburg position) on the other hand. This likewise includes the so-called ‘reclined seated position’, for which there is no date regarding its effects on haemodynamics and lung function. The *semi-seated position* refers to a position, in which—with bent hip and extended or bent knee joints—the upper body and the head of the patient are elevated by a certain degree as opposed to the flat-lying lower extremities (see Fig. 1).

What all modifications of the elevated upper body share in common is that the upper body is positioned above the level of the trunk, wherein the angle is at least 30° [75].

### Effect mechanisms of the backrest elevation

As a goal of the clinical trials, the gravitationally dependent effects of the elevated upper body position were studied. In this regard, the prevention of passive regurgitation (pulmonary aspiration of gastric contents) [63, 113] and the reduction of intracerebral blood volume (reducing intracranial pressure) were of primary focus. The remaining described effects of the elevated upper body position on haemodynamics (modified orthostatic reaction) and the pulmonary gas exchange (change of diaphragm position) were considered to be gravitationally dependent [24].

### Effects and impacts of backrest elevation on the lungs

#### Impacts on gastroesophageal reflux and pulmonary aspiration

The aspiration of secretion contaminated with bacteria in the gastrointestinal tract and the pharynx is generally perceived as a risk factor and trigger for the development of nosocomial and ventilator-associated pneumonia (VAP). Consequentially, measures that lead to decrease of gastrointestinal reflux and a reduction of the oropharyngeal secretion volume should accompany a lower incidence of nosocomial pneumonia and VAP [7, 142, 213] (evidence level 3).

Studies are available that have been conducted on patients with orotracheal intubation, who do not have known risk factors for gastroesophageal reflux. All patients were supplied with a nasogastric tube; some were fed enterally. Stress bleeding prophylaxis was conducted and the endotracheal cuff pressure was monitored (> 25 cm H_2_O). A 45° elevated upper body position in these patients lead to a delay of gastroesophageal reflux and to a decrease, though not a complete prevention, of pulmonary aspiration of pharyngeal secretion compared to a flat supine position [219, 290] (evidence level 2b).

In two prospective randomised trials [78, 117], a substantial reduction of VAP was observed through the application of a 45° backrest elevation compared to the supine position (evidence level 2b), however, both of these studies were heavily criticised with respect to their design and the method [213]. A small randomised pilot study observed a trend for reducing VAP with this position (evidence level 3) [154]. Further studies regarding feasibility and the effect of 45^°^ position [19, 20, 37, 214, 231, 248, 249, 250] revealed that precise compliance with the position in clinical practice is normally not feasible and a target angle of 45° could not be achieved (evidence level 2a). To improve practical implementation, numerous technical applications (angle measuring systems, trainings programmes for nursing staff) were recommended and implemented, which (with substantial effort) contributed to the increase of the precise execution [20, 37, 182, 311, 314] (evidence level 2b).

A systematic analysis and evaluation of the three randomised trials regarding the impact of the backrest elevation on VAP incidence by means of the Delphi method [213] did not reveal any clear evidence for the application of a 45° elevated upper body position due to the heterogeneity of the studies. Considering undesired accompanying effects, this expert consensus recommended that the elevated upper body position (20^°^–45^°^; more than 30^°^if possible) be used as a preferred position with reference to numerous limitations in ventilated patients (evidence level 2a). Despite the weakness of the Delphi recommendation, which is due to the weakness of the analysed studies, the guideline group supports this recommendation as it appears practical for clinical use and reflects the limited evidence.

**►23 The preferred principle position for intubated patients is the backrest elevation position of 20**°**–45**°**, preferably ≥ 30**^°^**, considering the limitations (evidence level 3, recommendation grade B).**

**For patients with elevated intracranial pressure, specific recommendations will be announced (see ► 27–29).**

#### Impacts on pulmonary gas exchange

Even in those with healthy lungs, anaesthesia and mechanical ventilation lead to a change of the regional ventilation with the development of atelectasis, particularly in the dorsal and diaphragm areas of the lungs. This effect is likely more distinctive in patients with increased intra-abdominal pressure (e.g. severe obesity, extensive surgical procedures on the abdomen, peritonitis) because the mobility of the diaphragm is limited and situated in cranial orientation. Even with ARDS, the impaired lung function leads to ventilation disorders and the formation of atelectasis. We must assume that actions for preventing diaphragm dislocation reduce the formation of atelectasis and thus contribute to an improvement of the gas exchange.

In one prospective crossover trial in 40 ARDS patients, the backrest elevation (20^°^–45^°^) leads to an increase of oxygenation in 32 % of the patients studied (> 20 % compared to the flat supine position) and to an increase of the lung volume [72] (evidence level 2b). In a similar crossover trial in 24 ventilated patients with difficult weaning, the 45^°^ position lead to a significant reduction of respiratory effort. Patients found the comfort level in this position to be the highest; no impact on the reduction of the weaning process was observed [74] (evidence level 2b).

In postoperative patients without ARDS, the semi-seated or sitting position lead to contradicting results with respect to the gas exchange compared to the supine position. In patients who were not characterised in more detail with pre-existing pulmonary diseases, the sitting position had no effect on capillary blood gases as opposed to the flat position regardless of age [212].

The effects of an intraoperative, semi-seated position on the gas exchange are also studied in neurosurgical patients [66]. The small amount of available data revealed an improvement of oxygenation in these patients. However, due to the fact that the intraoperative position was primarily determined by the surgery, a targeted, therapeutic application is not relevant (evidence level 4).

**►24 The elevated upper body position (20**°**–45**°**) may contribute to an improvement of oxygenation and the respiratory mechanics in patients with ARDS (evidence level 2b, recommendation grade B).**

**►25 Within the scope of the***difficult weaning***of mechanical ventilation (without the presence of COPD), the elevated upper body (45**°**) should be used to reduce respiratory effort and to increase the comfort level of the patient (evidence level 2b, recommendation grade B).**

### Backrest elevation in the case of obesity

In one prospective cohort study on 30 ventilated patients with obesity (BMI > 35 kg/m^2^), a significant reduction of expiratory flow limitation (= improvement of the gas flow) and a reduction of auto PEEP was revealed while in the sitting position (> 45°) compared to the lying position. These effects were not demonstrable in a control cohort (15 patients with BMI < 30 kg/m^2^) [173] (evidence level 2 b).

**►26 The flat supine position should be avoided in patients with severe obesity (evidence level 4, expert consensus). The backrest elevation position (**> **45**°**) may contribute to an improvement of the respiratory mechanics in ventilated patients with severe obesity (BMI** > **35 kg/m**^**2**^**) (evidence level 2b, recommendation grade 3). Regarding contraindications for the elevated upper body position**—**see ►28 und ►31**

### Impacts on other organ system

#### Intracerebral pressure (ICP) and cerebral perfusion pressure (CPP)

The elevated upper body position has been in treating ICP for a long time. Due to gravitationally dependent shifting, the cerebral blood and fluid volume are reduced and ICP decreases. However, the semi-seated position may also lead to an impact on haemodynamics and thus to a reduction of CPP. In patients with normal and elevated ICP, the elevated upper body position normally leads to a reduction of ICP depending on the angle [87]. An accompanying reduction of CPP can be observed more frequently with an elevated upper body position of 30° and greater. However, the breadth of the individual reaction through interactions with other parameters, such as ventilation pressure, sympathetic stimulation, haemodynamic function, volume status and level of sedation is vast and thus not predictable [44, 83, 89, 155, 180, 251, 313] (evidence level 3).

**►27 The application of an elevated upper body position of 15**°**–30**° **is sensible in patients with increased intracranial pressure and may contribute to a reduction of intracerebral pressure (evidence level 2b, recommendation grade B)**

**►28 A 45° backrest elevation cannot be recommended without limitation in patients with suspicion of increased intracranial pressure due to the fact that cerebral perfusion pressure can become critically degraded with an increasingly elevated position (evidence level 2b, recommendation grade B).**

**►29 With respect to the treatment of patients with elevated intracranial pressure, please refer to the S1 guideline***intracranial pressure***(AWMF registry no. 030/105, valid until 12/2015):***‘If possible, an elevated upper body position should be aimed for. The individually optimised upper body position should be regularly evaluated with ICP and CPP controls in the 0° (not in the case of the risk of aspiration or with ventilation), 15° and 30° position. Venous return flow should not be prevented by bending the head’***[**11**]***.*

#### Impacts on respiratory effort

Background: The most frequent postoperative complications after thoracic procedures are of a pulmonary nature caused by partial respiratory insufficiency as well as postoperative hypermetabolism with increased O_2_ consumption. Increased respiratory effort must be made by changing the lung volume particularly in patients with chronic obstructive pulmonary disease (COPD). Regarding the effects of the position, however, differences can be expected between patients with a chronic gas exchange disorder and those with acute exacerbation.

In patients following a thoracotomy, the semi-seated position resulted in a reduction of energy consumption without impacting haemodynamic function through a decrease of respiratory effort and oxygen consumption in the respiratory muscles [41] (evidence level 3b).

In noninvasively assisted ventilated COPD patients, the backrest elevation did not produce any changes in the respiratory volume, the respiratory pattern, respiratory effort or the gas exchange compared to the supine position or the lateral position [233]. The sitting position in patients with clinically significant dynamic distension, a deterioration of the activity of the diaphragm may occur to the extent that ventilation may be more effective in the supine position [81] (evidence level 4). Effects of the elevated upper body position on the pulmonary gas exchange and respiratory mechanism in ARDS patients and in patients with difficult weaning were described above.

**►30 In spontaneously breathing or noninvasively assisted breathing patients with COPD, positioning can occur pursuant to the individual request of the patient because the effects of a 45° elevated upper body position on respiratory effort have not been sufficiently documented (evidence level 4, recommendation grade 0).**

#### Impacts on haemodynamics

The semi-seated position may cause a reduction of cardiac output, blood pressure and peripheral oxygen supply due to a decrease of venous return to the heart.

In patients with ARDS, the semi-seated position or the anti-Trendelenburg position may cause the recognition of an existing volume deficit [140], which is treatable however through adequate volume substitution. The right ventricular function is not influenced by the elevated upper body position in the case of normovolaemia regardless of mechanical ventilation [312]. In contrast, a cardiac output decrease may occur in patients following abdominal procedures, which however may also be a recognition of persistent existing volume deficit [128]. Patients after a myocardial infarction individually demonstrate very differing changes of the haemodynamics as a reaction to the semi-seated position (evidence level 3b).

In a prospective randomised crossover study on 200 haemodynamically stable ventilated patients with different underlying disease [118], the position change of the upper body from 0° to 45° lead to a significant reduction of average arterial pressure and central venous oxygen saturation; this effect was less distinct at 30°. In a multivariate analysis, the following independent factors were identified for the development of hypotension within the scope of the 45° position: controlled ventilation (compared to augmented spontaneous ventilation), analgosedation, increased need for vasopressors, high PEEP and high Simplified Acute Physiology Score (SAPS-II) score (evidence level 1b).

**Under certain conditions, the backrest elevation (45**°**) may induce significant hypotension. Controlled ventilation (compared to augmented spontaneous ventilation), continuous analgosedation, an increased need for vasopressors, a high PEEP and a high SAP-II score are considered to be risk factors for this (evidence level 2b).**

**►31 The elevated upper body position of 45**° **is not recommended in the presence of this/these constellation(s). A maximum backrest elevation of 30**° **should be conducted in these patients (evidence level 2b, recommendation grade B).**

#### Elevated upper body position and intra-abdominal pressure

Multiple studies [189, 247, 262, 296] described an increase of intra-abdominal pressure (diverted through the bladder) within the scope of an increasing elevated upper body position in cohort studies on intensive care patients (37–120 patients), wherein no critical values (> 15 mm Hg) were achieved at the 45° position (evidence level 3). No patients with an existing abdominal disorder or verifiable intra-abdominal pressure increase were found in these groups. An overview and evaluation of these studies [156] critically dealt with the significance of measuring bladder pressure within the scope of the elevated upper body position.

**The elevated upper body position with bending of the hip may affect an increase in intra-abdominal pressure (diverted through the bladder) (evidence level 3).**

**►32 In patients with abdominal disease or severe obesity, the anti-Trendelenburg position without bending of the hip should be preferred for the elevated upper body position (evidence level 3, recommendation grade B).**

#### Elevated upper body position and the occurrence of decubitus ulcers in proximal tissue

In a prospective crossover study with a variation of the upper body position (0°–75°) the pressure on proximal tissue (in the sacral area) was measured in healthy test persons [232]. A significant and critical increase (> 32 mm Hg) was revealed in the sacral area starting at an elevated upper body position of 45°. A significant, but less distinct pressure increase was also measured in the 30° position (evidence level 3). There are no studies for ventilated or critically ill intensive care patients.

**The elevated upper body position** > **30**° **with bending of the hip can lead to a critical increase of the pressure on the skin in the sacral area.**

**►33 It is recommended with critically ill intensive care patients to reduce bending of the hip while in the elevated upper body position using the anti-Trendelenburg position (evidence level 3, recommendation grade 0).**

## Unsuitable positions in intensive care patients

Two positions, namely the supine position and the Trendelenburg position are particularly unsuitable for long-term application in critically ill patients and should only be applied in special situations, for example cardiopulmonary resuscitation, volume deficit shock, insertion of central venous catheters. However, the positioning wish of the patient must also be taken into consideration when positioning.

### Flat supine position

#### Definition

The supine position refers to a position, in which the patient lies flat and horizontally on his back.

If someone with a normal weight lies in the flat supine position, an increased venous return flow to the heart will occur. Cardiac output, pulmonary blood flow and arterial blood pressure increase, the functional residual capacity (FRC) decreases, the diaphragm is compromised by the abdomen and limited in its mobility. Anaesthesia, analgosedation or muscle relaxants increase the undesired effects [239]. The reduced FRC will also lead to the collapse of small respiratory tracts, to the formation of atelectasis and to a limited pulmonary gas exchange [69].

The flat supine position can be dangerous particularly for obese patients. It can lead to acute heart failure, respiratory arrest and pronounced pulmonary gas exchange disorders [165, 172, 292, 315]. Death in the extremely obese due to the flat supine position is referred to as ‘*obesity supine death syndrome’* [292].

Expiratory flow impediments, the development of an auto PEEP as well as a collapse of small respiratory tracts occurred regularly in mechanically ventilated obese patients in the flat supine position if an external ZEEP (zero endexpiratory pressure) or too low of a PEEP level was selected [174].

If there is a combination of COPD and obesity, a tracheomalacia can only be expected in rare cases (3 %) based on a differential diagnosis, which becomes symptomatic in the flat supine position [133, 165].

**►34 The flat supine position should not be applied in critically ill patients due to the numerous unfavourable effects on haemodynamics and pulmonary gas exchange (evidence level 3, recommendation grade B).**

**If the application of the flat supine position is absolutely necessary for special medical or nursing measures, it should be limited to the shortest possible period (evidence level 4, recommendation grade A).**

### Trendelenburg position

#### Definition

The Trendelenburg position is a variation of the flat supine position, in which the head is at the lowest position of the body through the inclined positioning of the bed. It was used regularly starting in 1880 by the surgeon, Friedrich Trendelenburg (*1844, †1924), during urological and gynaecological procedures and remained widely popular in the following decades [196, 197].

The Trendelenburg position is an extreme strain on the respiratory and cardiovascular system of the critically ill patient. Blood is channelled from the lower parts of the body toward the heart and causes a right heart overload. The abdominal organs and—in the case of the obese—the abdominal fat masses press the diaphragm upward and compromise the lungs. The Trendelenburg position leads to a variety of physiological/pathophysiological changes: an increase in the stroke volume of the heart, the pressure on the central veins and pulmonary arteries, the resistance of the vascular system, the right and left ventricular end systolic volume index, cardiac output and intrathoracic blood volume as well as to reduced cerebral blood flow, to reduced systemic oxygenation and an increase of arterial carbon dioxide partial pressure. The FRC decreases; atelectasis formation occurs [129].

The Trendelenburg position is the most hazardous position for the obese [191, 264]. It should not be applied in spontaneously breathing, awake, obese patients. For anaesthesiological and intensive care interventions (e.g. applying a central venous catheter, etc.), the obese patient should not be placed in the Trendelenburg position.

**►35 The Trendelenburg position should not be applied in critically ill patients due to numerous unfavourable effects on haemodynamics, pulmonary gas exchange and the respiratory system (evidence level 3, recommendation grade B).**

**If the application of the Trendelenburg position is absolutely necessary for special medical or nursing measures, it should be limited to a brief period (evidence level 4, recommendation grade A).**

**►36 The Trendelenburg position should principally be avoided in obese patients (evidence level 3a, recommendation grade A).**

## Early mobilisation

### Definition of mobilisation

The term *mobilisation* describes measures involving the patient, which introduce and/or assist passive or active movement exercises and which aim to promote and/or maintain mobility. In contrast, positioning refers to the change of bodily positions with the goal of influencing gravity-related effects [3, 121, 153, 176].

**Early mobilisation refers to the beginning of mobilisation within 72 h after admittance to intensive care.**

### Elements of mobilisation

Methods for mobilisation are classified in three areas: passive mobilisation, assisted active mobilisation and active mobilisation [3, 9, 80, 84, 85, 130, 176, 153, 230, 258, 259, 319]. These three areas can be structured as follows:

*Passive mobilisation:*

- Passive motions of all extremities in all physiological directions
- Passive *cycling* (bed pedal exerciser)
- Passive vertical mobilisation (tilting table, standing frame)
- Passive transfer to rehabilitation chair

*Assisted active mobilisation:*

- Active movement exercises in the supine position with manual support
- Independent mobilisation in bed (sitting down upright, turning)
- Balance training
- Assisted *cycling*

*Active mobilisation:*

- Sitting on the edge of the bed, torso control
- Active mobilisation to the status
- Attempting to stand up, walking exercises while standing
- Walking with and without walking aids
- Active cycling
- Isotonic movement exercises with walking aids

### Goals of mobilisation

The general goals of mobilisation are to promote and maintain mobility as well as to prevent and/or reduce the effects of immobilisation. *Immobilisation* refers to the idle position of the bodily parts or the entire body for the purpose of treatment or for rest (bed rest). Undesired effects of immobilisation are a general *deconditioning*, the development of a weakness, rapid fatigue and atrophy of the muscular respiratory pumps and the skeletal muscles, the development of psycho-cognitive deficits and delirium, the emergence of positioning-related skin and soft tissue damage as well as the reduction of haemodynamic responsiveness [47, 179].

The specific *goals of mobilisation* consist in improving/maintaining skeletal and respiratory muscle function, increasing haemodynamic responsiveness, improving central and peripheral perfusion and muscle metabolism, increasing cognitive competence and mental wellbeing, reducing incidence and duration of delirium, reducing positioning-related skin ulcers and—compared to patients, who were not mobilised early—improving the subsequent health-related quality of life [3, 9, 121, 153, 176].

### Effects of early mobilisation on treatment success

When recording and assessing the effects of early mobilisation on the outcome, various relevant parameters are included. These include bodily function outcomes, peripheral muscle strength and function of the muscular respiratory pump, neurocognitive competence, ventilator-free days, ICU stay, hospitalisation, mortality, quality of life and discharge from the hospital.

The following prospective randomised trials are suitable for an analysis: Morris et al*.* [199] discovered a substantially shorter treatment period in the intensive care unit and in the hospital as well as a trend for shorter treatment costs in early mobilised patients. In Burtin et al. [48], significantly higher muscle strength in the quadriceps as well as a significantly higher state of functional independence (SF-36) after discharge was observed following early mobilisation. Schweickert et al. [259] describe a substantially longer walking distance after intensive care treatment, a significantly higher Barthel index, a significantly higher state of functional independence (SF-36), a shorter ventilation period during intensive care treatment and a trend toward greater probability of discharge in the early mobilisation group (all—evidence level 2b). Other prospective randomised trials with limited quality [35, 52, 55, 64, 82, 208] underscore other outcome findings—Chen et al. [ 55] detected a lower one-year mortality rate among a very small group of patients in the early mobilisation group. Cuesy et al. [64] observed a significant reduction of incidences of nosocomial pneumonia among stroke patients in the patient group that experienced passive mobilisation at an early stage (‘turn-mob’). Nava et al*.* [208] studied the effect of early mobilisation in patients with COPD during a six-minute walk. The patients of the group with early mobilisation walked a significantly longer distance. Bezbaruah et al. [35] recognised a substantially shorter ICU treatment duration in early mobilisation patients in a small study (all—evidence level 3).

**►37 In principle, early mobilisation should be conducted in all patients treated in intensive care, for whom no exclusion criteria apply (evidence level 2b, recommendation grade A).**

### Patient-related requirements/suitability for mobilisation

Patient-related requirements and suitability for mobilisation was reviewed in multiple observational studies [15, 40, 56, 103, 121, 139, 152, 168, 181, 201, 317]. Bourdin et al. [40] systematically compiled 275 interventions, for which 33 % of ventilated patients were mobilised. Getting up out of the chair (56 % of actions) was linked to a substantial reduction in heart and respiratory rate; average arterial blood pressure and arterial oxygen saturation (pulse oxymetry) remained unchanged. Continued standing (25 %) and walking (11 %) resulted in a heart and respiratory rate increase and a significant decrease of arterial oxygen saturation (evidence level 2b). Kasotakis et al. [152] presented a *surgical intensive care unit optimal mobility score*, which captured the exclusion of serious organ function disorders and the suitability for mobilisation prior to mobilisation. In one prospective study, this score proved to be better suited—compared to other general scores (comorbidity index, APACHE)—to determine suitability for mobilisation (evidence level 3).

**►38 Regarding early mobilisation, the following requirements should be present or established:**

- **Customised, score-controlled (e.g. RASS) symptom control of pain, fear, agitation and delirium according to the S3 guideline revision ‘Analgosedation’***(German Society of Anesthesia and Intensive Care)*
- **Sufficient respiratory reserve**
- **Sufficient cardiovascular reserve**

**The following serve as reference points for this: average arterial blood pressure** > **65 or** < **110 mm Hg, systolic blood pressure** < **200 mm Hg, heart rate** > **40 or** < **130/min, arterial oxygen saturation (pulse oxymetry) ≥ 88 %, no higher-dosage vasopressor therapy.**

**If cardiopulmonary instability develops during ongoing mobilisation, the exercise unit should be discontinued until stabilisation returns or conducted to an adapted extent (evidence level 2b, recommendation grade A).**

### Criteria for checking the feasibility/contraindications or cancellation criteria for (early) mobilisation

Clearly defined exclusion criteria for early mobilisation are not designated in the literature. However, the requirement for mobilisation should be evaluated for certain acute situations in a symptom-adapted manner. The following examples are described in the literature [9, 97, 109, 150, 159, 218, 268, 269, 281, 289, 318]:

- increased intracranial pressure
- active bleeding
- acute myocardial ischaemia
- agitated delirium

**►39 The decision to conduct limited forms of mobilisation (passive or active with assistance) with the specified relative contraindications should be considered in individual cases in light of the benefits and risks (evidence level 2b, recommendation grade A).**

### Preparation/monitoring

The preparation for mobilisation and monitoring of the patient during the action is described in various observational studies or randomised trials [15, 40, 103, 139, 152, 168, 181, 201, 317]. This includes the information of the patient, the provisioning of sufficient staff and the securing/extension of structures of the mechanical respiratory tract, the infusion lines or other drainages as well as the monitoring of vital parameters during the procedure.

**►40 The preparation of early mobilisation comprises the information of the patient, the provisioning of sufficient staff and the securing/extension of structures of the mechanical respiratory tract, the infusion lines or other drainages. During mobilisation, the heart rate, blood pressure and arterial oxygen saturation should be continuously/closed recorded for the monitoring of the vital parameters (evidence level 2b, recommendation grade A).**

**►41 In ventilated patients, the ventilation parameters should be continuously featured (tidal volume, inspiratory pressure, respiratory rate, respiratory minute volume; in the case of invasively ventilated patients capnometry) (evidence level 3, recommendation grade B)**

### Duration and intensity of mobilisation

In the prospective randomised studies [84, 85, 121, 130, 192, 270] deemed suitable for the meta-analyses [3, 121, 176], early mobilisation was started within 72 h after admittance to intensive care with a gradual increase. The actions were conducted on average or at least 20 min twice daily.

**►42 Treatment should begin no later than 72 h after admittance to intensive care and be conducted twice daily with a duration of at least 20 min for the length of stay in intensive care. A gradual approach should be aimed for starting with passive mobilisation** (Table 5)**. In this regard, the development of an algorithm specific to a unit or hospital is recommended (evidence level 3, recommendation grade B)**

**Table 5:** Components for an ‘early mobilisation’ algorithm. The essential initial conditions of the patient, the aid to be used, the suitable procedure and formulation of objectives are listed without clear allocation. The allocation is the result of available staff resources and aids of the respective intensive therapy unit. The stated actions are examples without claim of completeness. Further information can be found at the *German Early Mobilisation Network* (www.frühmobilisierung.de)

Patient	Aid	Method	Goal
Limited vigilance (RASS ≥ − 3)	–	Passive motion	Prophylaxis of joint contractions and muscle loss
		Passive cycling	
Increasing vigilance (RASS − 3 to − 1)	Mobilisation chair Tilting table	Activated sitting in bed	Prophylaxis of ‘deconditioning’ and delirium
		Moving the extremities against gravity	
		Vertical mobilisation	
		Passive cycling	
		(Passive) transfer to mobilisation chair	
Return of vigilance (RASS ≥ 0)	Mobilisation chair	Active cycling	Prophylaxis of *deconditioning*, delirium and pulmonary function disorders
		(Active) transfer to mobilisation chair	
No serious haemodynamic instability	Mobilisation chair	Standing in front of the bed	Prophylaxis of *deconditioning*, delirium and pulmonary function disorders
		Walking exercises while standing	
	Walking aids	Walking with and without walking aid	Prophylaxis of *deconditioning*, delirium and pulmonary function disorders

*RASS* Richmond Agitation Sedation Scale.

### Safety aspects/complications and cancellation criteria within the scope of mobilisation

The following complications are described in individual cases within the scope of mobilisation: orthostatic dysregulation, patient fall, disconnection of catheters/airway, cardiac dysrhythmias, respiratory fatigue/dyspnoea and agitation/stress [103, 150, 268, 269]. In a systematic of overall four studies, no serious complications were detected within the scope of mobilisation, which involved further intervention other than the termination of the action [150]. Mobilisation should be cancelled in the event of the following signs of intolerance: SaO_2_ < 88 %, heart rate increase > 20 % or heart rate < 40 or > 130/min, newly occurring cardiac dysrhythmias, systolic blood pressure > 180 mm Hg or mean blood pressure < 65 mm Hg or > 110 mm Hg (evidence level 2b). Overall, the occurrence of undesired events provided with an incidence of 1.1–4.4 %.

**In consideration for patient-related requirements and potential exclusion criteria as well as compliance with preparation measures, early mobilisation presents a safe and uncomplicated method.**

**►43 Cancellation of mobilisation is recommended in the event of the following vital parameter changes: SaO**_**2**_ < **88 %, heart rate increase** > **20 % or heart rate** < **40 or** > **130/min, new cardiac dysrhythmias, systolic blood pressure** > **180 mm Hg or mean blood pressure** < **65 mm Hg or** > **110 mm Hg (evidence level 2b, recommendation grade A).**

### Structure/organisation/personnel/expense/protocol

Early mobilisation represents an interdisciplinary, targeted approach in improving the results of intensive care. The establishment of a concept for this tiered, specific approach in consideration for safety aspects was classified as beneficial in multiple publications [9, 16, 64, 67, 80, 84, 130, 192, 230, 259, 319]. A *standard of care* is recommended [230], which enables a tiered, customised increase of mobilisation in four phases, particularly in ventilated patients [259] as well. Appropriate personnel and spatial requirements are integrated in this *standard of care*. In a prospective observation study, the regular integration of a physiotherapist in early mobilisation proved to have a better effect on outcome parameters compared to early mobilisation without physiotherapeutic aid [131, 270] (evidence level 2b).

**►44 A protocol-based approach is recommended for implementing early mobilisation. Active mobilisation should be conducted by at least two qualified staff members; a physiotherapist should be regularly integrated. Sufficient spatial requirements and resources should be kept.**

**►45 Early mobilisation should be incorporated into a set of measures, which includes the strategy for adapted symptom monitoring of pain, fear, agitation and delirium, as well as for the daily assessment of spontaneous breathing (evidence level 2b, recommendation level A).**

## Outlook: early muscle activation through electrical muscle stimulation

A few observational studies describe that the application of electrical muscle stimulation in critically ill intensive care patients has a positive effect on the preservation of muscle mass and muscle strength [112, 151, 253, 246, 308]. Very different patient groups were studied at various times while in intensive care in these studies. Comparative, randomised studies do not exist. It is currently unclear, which patients benefited at which point from electrical muscle stimulation while in intensive care [305].

**Fig. 2:**
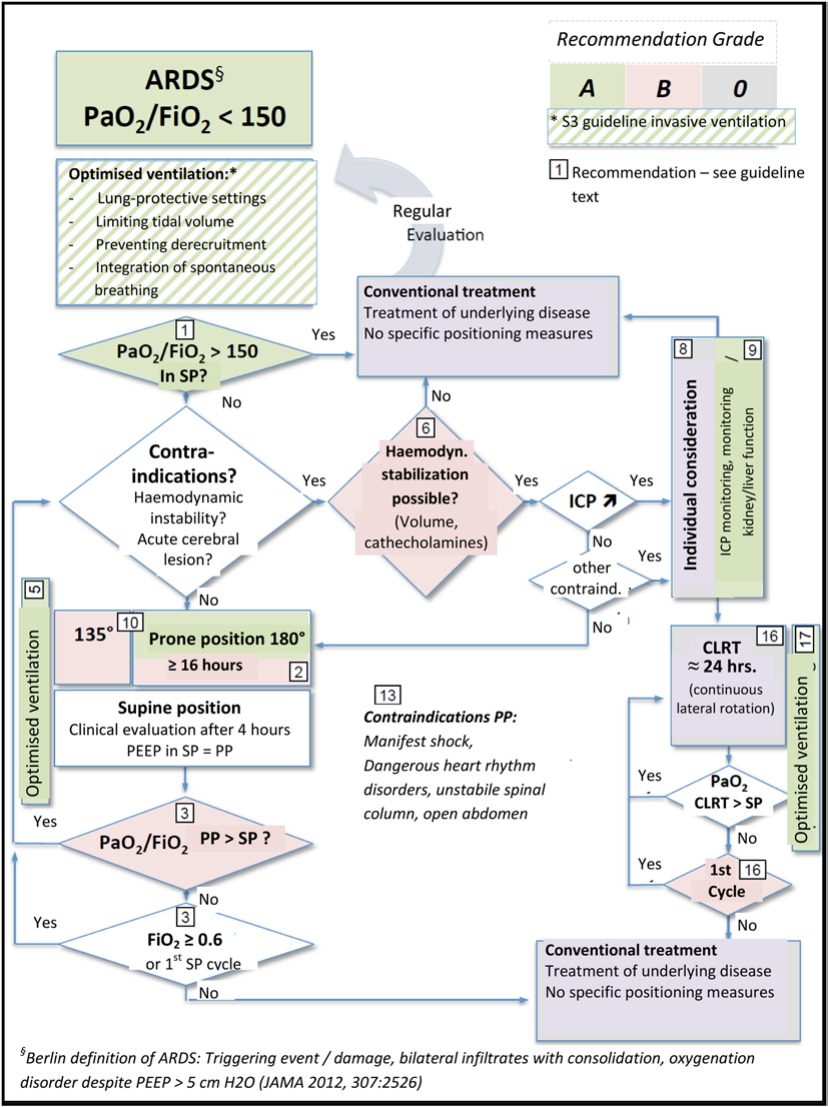
Algorithm for positioning therapy in intensive care. *SP* supine position, *PP* prone position, *ICP* intracranial pressure, *CLRT* continuous lateral rotation therapy, *ARDS* acute respiratory distress syndrome, *PEEP* positive end-expiratory pressure

At the moment, no recommendation for the use of electrical muscle stimulation in intensive care patients can be expressed due to the incomplete data (Fig. 2).

## References

[1] Abroug F, Ouanes-Besbes L, Dachraoui F (2011). An updated study-level meta-analysis of randomised controlled trials on proning in ARDS and acute lung injury. Crit Care.

[2] Abroug F, Ouanes-Besbes L, Elatrous S (2008). The effect of prone positioning in acute respiratory distress syndrome or acute lung injury: a meta-analysis. Areas of uncertainty and recommendations for research. Intensive Care Med.

[3] Adler J, Malone D (2012). Early mobilization in the intensive care unit: a systematic review. Cardiopulm Phys Ther J.

[4] Ahrens T, Kollef M, Stewart J (2004). Effect of kinetic therapy on pulmonary complications. Am J Crit Care.

[5] Albert RK, Hubmayr RD (2000). The prone position eliminates compression of the lungs by the heart. Am J Respir Crit Care Med.

[6] Albert RK, Leasa D, Sanderson M (1987). The prone position improves arterial oxygenation and reduces shunt in oleic-acid-induced acute lung injury. Am Rev Respir Dis.

[7] Alexiou VG, Ierodiakonou V, Dimopoulos G (2009). Impact of patient position on the incidence of ventilator-associated pneumonia: a meta-analysis of randomized controlled trials. J Crit Care.

[8] Alsaghir AH, Martin CM (2008). Effect of prone positioning in patients with acute respiratory distress syndrome: a meta-analysis. Crit Care Med.

[9] Amidei C (2012). Mobilisation in critical care: a concept analysis. Intensive Crit Care Nurs.

[10] Anzueto A, Peters JI, Seidner SR (1997). Effects of continuous bed rotation and prolonged mechanical ventilation on healthy, adult baboons. Crit Care Med.

[11] Arbeitsgemeinschaft der Wissenschaftlichen Medizinischen Fachgesellschaften (AWMF) (2012)—Leitlinie Intrakranieller Druck (ICP)—Entwicklungsstufe: S1, Stand: September 2012, Gültig bis: Dezember 2015, AWMF-Registernummer: 030/105

[12] Aries MJH, Aslan A, Elting JWJ (2012). Intra-arterial blood pressure reading in intensive care unit patients in the lateral position. J Clin Nurs.

[13] Athota KP, Millar D, Branson RD, Tsuei BJ (2014). A practical approach to the use of prone therapy in acute respiratory distress syndrome. Expert Rev Respir Med.

[14] Badia JR, Sala E, Rodriguez-Roisin R (1998). Positional changes and drug interventions in acute respiratory failure. Respirology.

[15] Bahadur K, Jones G, Ntoumenopoulos G (2008). An observational study of sitting out of bed in tracheostomised patients in the intensive care unit. Physiotherapy.

[16] Balas MC (2000). Prone positioning of patients with acute respiratory distress syndrome: applying research to practice. Crit Care Nurse.

[17] Balas M, Buckingham R, Braley T (2013). Extending the ABCDE bundle to the post-intensive care unit setting. J Gerontol Nurs.

[18] Ball C, Adams J, Boyce S (2001). Clinical guidelines for the use of the prone position in acute respiratory distress syndrome. Intensive Crit Care Nurs.

[19] Ballew C, Buffmire MV, Fisher C (2011). Factors associated with the level of backrest elevation in a thoracic cardiovascular intensive care unit. Am J Crit Care.

[20] Balonov K, Miller AD, Lisbon A (2007). A novel method of continuous measurement of head of bed elevation in ventilated patients. Intensive Care Med.

[21] Banasik JL, Emerson RJ (1996). Effect of lateral position on arterial and venous blood gases in postoperative cardiac surgery patients. Am J Crit Care.

[22] Banasik JL, Emerson RJ (2001). Effect of lateral positions on tissue oxygenation in the critically ill. Heart Lung.

[23] Baraka A, Moghrabi R, Yazigi A (1987). Unilateral pulmonary oedema/atelectasis in the lateral decubitus position. Anaesthesia.

[24] Baydur A, Sassoon CS, Carlson M (1996). Measurement of lung mechanics at different lung volumes and esophageal levels in normal subjects: effect of posture change. Lung.

[25] Bein T, Metz C, Eberl P (1994). Acute pulmonary and cardiovascular effects of continuous axial rotation (kinetic therapy) in respiratory failure. Schweiz Med Wochenschr.

[26] Bein T, Metz C, Keyl C (1996). Effects of extreme lateral posture on hemodynamics and plasma atrial natriuretic peptide levels in critically ill patients. Intensive Care Med.

[27] Bein T, Ploner F, Ritzka M (2010). No change in the regional distribution of tidal volume during lateral posture in mechanically ventilated patients assessed by electrical impedance tomography. Clin Physiol Funct Imaging.

[28] Bein T, Reber A, Metz C (1998). Acute effects of continuous rotational therapy on ventilation-perfusion inequality in lung injury. Intensive Care Med.

[29] Bein T, Ritzka M, Schmidt F (2007). Positioning therapy in intensive care medicine in Germany. Results of a national survey. Anaesthesist.

[30] Bein T, Sabel K, Scherer A (2004). Comparison of incomplete (135 degrees) and complete prone position (180 degrees) in patients with acute respiratory distress syndrome. Results of a prospective, randomised trial. Anaesthesist.

[31] Bein T, Zimmermann M, Schiewe-Langgartner F (2012). Continuous lateral rotational therapy and systemic inflammatory response in posttraumatic acute lung injury: results from a prospective randomised study. Injury.

[32] Bein T, Reber A, Ploner F (2007) Continuous axial rotation and pulmonary fluid balance in acute lung injury. Clin Intensive Care 11:307–310

[33] Beitler JR, Shaefi S, Montesi SB (2014). Prone positioning reduces mortality from acute respiratory distress syndrome in the low tidal volume era: a meta-analysis. Intensive Care Med.

[34] Beuret P, Carton M, Nourdine K (2002). Prone position as prevention of lung injury in comatose patients: a prospective, randomized, controlled study. Intensive Care Med.

[35] Bezbaruah P, Swaminathan N, D C (2012). Effect of graded early mobilization versus routine physiotherapy on the length of intensive care unit stay in mechanically ventilated patients: a randomized controlled study. Intern J Health Allied Sciences.

[36] Blanch L, Mancebo J, Perez M (1997). Short-term effects of prone position in critically ill patients with acute respiratory distress syndrome. Intensive Care Med.

[37] Bloos F, Muller S, Harz A (2009). Effects of staff training on the care of mechanically ventilated patients: a prospective cohort study. Br J Anaesth.

[38] Bodmann KF, Lorenz J, Bauer TT (2003). Nosokomiale Pneumonie: Prävention, Diagnostik und Therapie. Chemother J.

[39] Borelli M, Lampati L, Vascotto E (2000). Hemodynamic and gas exchange response to inhaled nitric oxide and prone positioning in acute respiratory distress syndrome patients. Crit Care Med.

[40] Bourdin G, Barbier J, Burle J (2010). The feasibility of early physical activity in intensive care unit patients: a prospective observational one-center study. Respir Care.

[41] Brandi LS, Bertolini R, Janni A (1996). Energy metabolism of thoracic surgical patients in the early postoperative period. Effect of posture. Chest.

[42] Breiburg AN, Aitken L, Reaby L (2000). Efficacy and safety of prone positioning for patients with acute respiratory distress syndrome. J Adv Nurs.

[43] Brettner F, Tsekos E, Schmidt A (1999). Splenic rupture as a complication of ventilation in the prone position and pneumococcal sepsis as a late complication. Anasthesiol Intensivmed Notfallmed Schmerzther.

[44] Brimioulle S, Moraine JJ, Norrenberg D (1997). Effects of positioning and exercise on intracranial pressure in a neurosurgical intensive care unit. Phys Ther.

[45] Broccard A, Shapiro RS, Schmitz LL (2000). Prone positioning attenuates and redistributes ventilator-induced lung injury in dogs. Crit Care Med.

[46] Broccard AF, Shapiro RS, Schmitz LL (1997). Influence of prone position on the extent and distribution of lung injury in a high tidal volume oleic acid model of acute respiratory distress syndrome. Crit Care Med.

[47] Brower RG (2009). Consequences of bed rest. Crit Care Med.

[48] Burtin C, Clerckx B, Robbeets C (2009). Early exercise in critically ill patients enhances short-term functional recovery. Crit Care Med.

[49] Cason CL, Holland CL, Lambert CW (1990). Effects of backrest elevation and position on pulmonary artery pressures. Cardiovasc Nurs.

[50] Chan M, Jensen L (1992). Positioning effects on arterial oxygen and relative pulmonary shunt in patients receiving mechanical ventilation after CABG. Heart Lung.

[51] Chandy D, Sahityani R, Aronow WS (2007). Impact of kinetic beds on the incidence of atelectasis in mechanically ventilated patients. Am J Ther.

[52] Chang MY, Chang LY, Huang YC (2011). Chair-sitting exercise intervention does not improve respiratory muscle function in mechanically ventilated intensive care unit patients. Respir Care.

[53] Chang SC, Chang HI, Shiao GM (1993). Effect of body position on gas exchange in patients with unilateral central airway lesions. Down with the good lung?. Chest.

[54] Chatte G, Sab JM, Dubois JM (1997). Prone position in mechanically ventilated patients with severe acute respiratory failure. Am J Respir Crit Care Med.

[55] Chen S, Su C, Wu Y (2011). Physical training is beneficial to functional status and survival in patients with prolonged mechanical ventilation. J Formos Med Assoc.

[56] Cheung N, To K, Dickinson S (2012). Early mobility (EM) protocol in a surgical intensive care unit (SICU): impact on risk factors for pressure ulcer (PU) development. Crit Care Med.

[57] Chipman JG, Taylor JH, Thorson M (2006). Kinetic therapy beds are associated with more complications in patients with thoracolumbar spinal column injuries. Surg Infect.

[58] Chiumello D, Cressoni M, Racagni M (2006). Effects of thoraco-pelvic supports during prone position in patients with acute lung injury/acute respiratory distress syndrome: a physiological study. Crit Care.

[59] Choe KH, Kim YT, Shim TS (2000). Closing volume influences the postural effect on oxygenation in unilateral lung disease. Am J Respir Crit Care Med.

[60] Clemmer TP, Green S, Ziegler B (1990). Effectiveness of the kinetic treatment table for preventing and treating pulmonary complications in severely head-injured patients. Crit Care Med.

[61] Colmenero-Ruiz M, Pola-Gallego de Guzman D, Jimenez-Quintana MM (2001). Abdomen release in prone position does not improve oxygenation in an experimental model of acute lung injury. Intensive Care Med.

[62] Cornejo RA, Diaz JC, Tobar EA (2013). Effects of prone positioning on lung protection in patients with acute respiratory distress syndrome. Am J Respir Crit Care Med.

[63] Craven DE, Steger KA (1998). Ventilator-associated bacterial pneumonia: challenges in diagnosis, treatment, and prevention. New Horiz.

[64] Cuesy PG, Sotomayor PL, Pina JOT (2010). Reduction in the incidence of poststroke nosocomial pneumonia by using the “turn-mob” program. J Stroke Cerebrovasc Dis.

[65] Curley MA, Thompson JE, Arnold JH (2000). The effects of early and repeated prone positioning in pediatric patients with acute lung injury. Chest.

[66] Dalrymple DG, MacGowan SW, MacLeod GF (1979). Cardiorespiratory effects of the sitting position in neurosurgery. Br J Anaesth.

[67] Davis J, Crawford K, Wierman H (2013). Mobilization of ventilated older adults. J Geriatr Phys Ther.

[68] Davis K, Johannigman JA, Campbell RS (2001). The acute effects of body position strategies and respiratory therapy in paralyzed patients with acute lung injury. Crit Care.

[69] Dean E (1985). Effect of body position on pulmonary function. Phys Ther.

[70] deBoisblanc BP, Castro M, Everret B (1993). Effect of air-supported, continuous, postural oscillation on the risk of early ICU pneumonia in nontraumatic critical illness. Chest.

[71] Delaney A, Gray H, Laupland KB (2006). Kinetic bed therapy to prevent nosocomial pneumonia in mechanically ventilated patients: a systematic review and meta-analysis. Crit Care.

[72] Dellamonica J, Lerolle N, Sargentini C (2013). Effect of different seated positions on lung volume and oxygenation in acute respiratory distress syndrome. Intensive Care Med.

[73] Deniz MN, Erakgun A, Sertoz N (2013). The effect of head rotation on intraocular pressure in prone position: a randomized trial. Braz J Anesthesiol.

[74] Deye N, Lellouche F, Maggiore SM (2013). The semi-seated position slightly reduces the effort to breathe during difficult weaning. Intensive Care Med.

[75] Dillon A, Munro CL, Grap MJ (2002). Nurses. Am J Crit Care.

[76] Doering LV (1993). The effect of positioning on hemodynamics and gas exchange in the critically ill: a review. Am J Crit Care.

[77] Dolovich M, Rushbrook J, Churchill E (1998). Effect of continuous lateral rotational therapy on lung mucus transport in mechanically ventilated patients. J Crit Care.

[78] Drakulovic MB, Torres A, Bauer TT (1999). Supine body position as a risk factor for nosocomial pneumonia in mechanically ventilated patients: a randomised trial. Lancet.

[79] Dreyfuss D, Djedaini K, Lanore JJ (1992). A comparative study of the effects of almitrine bismesylate and lateral position during unilateral bacterial pneumonia with severe hypoxemia. Am Rev Respir Dis.

[80] Drolet A, DeJuilio P, Harkless S (2013). Move to improve: the feasibility of using an early mobility protocol to increase ambulation in the intensive and intermediate care settings. Phys Ther.

[81] Druz WS, Sharp JT (1982). Electrical and mechanical activity of the diaphragm accompanying body position in severe chronic obstructive pulmonary disease. Am Rev Respir Dis.

[82] Dull JL, Dull WL (1983). Are maximal inspiratory breathing exercises or incentive spirometry better than early mobilization after cardiopulmonary bypass?. Phys Ther.

[83] Durward QJ, Amacher AL, Del Maestro RF (1983). Cerebral and cardiovascular responses to changes in head elevation in patients with intracranial hypertension. J Neurosurg.

[84] Engel HJ, Needham DM, Morris PE (2013). ICU early mobilization: from recommendation to implementation at three medical centers. Crit Care Med.

[85] Engels PT, Beckett AN, Rubenfeld GD (2013). Physical rehabilitation of the critically ill trauma patient in the ICU. Crit Care Med.

[86] Erhard J, Waydhas C, Ruchholtz S (1998). Effect of kinetic therapy on the treatment outcome in patients with post-traumatic lung failure. Unfallchirurg.

[87] Fan J (2004). Effect of backrest position on intracranial pressure and cerebral perfusion pressure in individuals with brain injury: a systematic review. J Neurosci Nurs.

[88] Farag E, Sessler DI, Kovaci B (2012). Effects of crystalloid versus colloid and the α-2 agonist brimonidine versus placebo on intraocular pressure during prone spine surgery: a factorial randomized trial. Anesthesiology.

[89] Feldman Z, Kanter MJ, Robertson CS (1992). Effect of head elevation on intracranial pressure, cerebral perfusion pressure, and cerebral blood flow in head-injured patients. J Neurosurg.

[90] Ferguson ND, Fan E, Camporota L (2012). The Berlin definition of ARDS: an expanded rationale, justification, and supplementary material. Intensive Care Med.

[91] Fernandez R, Trenchs X, Klamburg J (2008). Prone positioning in acute respiratory distress syndrome: a multicenter randomized clinical trial. Intensive Care Med.

[92] Fichele S, Woodhouse N, Swift AJ (2004). MRI of helium-3 gas in healthy lungs: posture related variations of alveolar size. J Magn Reson Imaging.

[93] Fink MP, Helsmoortel CM, Stein KL (1990). The efficacy of an oscillating bed in the prevention of lower respiratory tract infection in critically ill victims of blunt trauma. A prospective study. Chest.

[94] Fischer JA (2000). How to promote pulmonary health with kinetic therapy. Nurs Manage.

[95] Fishman AP (1981). Down with the good lung. N Engl J Med.

[96] Flaatten H, Aardal S, Hevroy O (1998). Improved oxygenation using the prone position in patients with ARDS. Acta Anaesthesiol Scand.

[97] Flanders SA, Harrington L, Fowler RJ (2009). Falls and patient mobility in critical care: keeping patients and staff safe. Adv Crit Care.

[98] Fleegler B, Grimes C, Anderson R (2009). Continuous lateral rotation therapy for acute hypoxemic respiratory failure: the effect of timing. DCCN.

[99] Fletcher SJ (2006). The effect of prone ventilation on intra-abdominal pressure. Clin Intensive Care.

[100] Fridrich P, Krafft P, Hochleuthner H (1996). The effects of long-term prone positioning in patients with trauma-induced adult respiratory distress syndrome. Anesth Analg.

[101] Gainnier M, Michelet P, Thirion X (2003). Prone position and positive end-expiratory pressure in acute respiratory distress syndrome. Crit Care Med.

[102] Galiatsou E, Kostanti E, Svarna E (2006). Prone position augments recruitment and prevents alveolar overinflation in acute lung injury. Am J Respir Crit Care Med.

[103] Garzon-Serrano J, Ryan C, Waak K (2011). Early mobilization in critically ill patients: patients. PM R.

[104] Gattinoni L, Pelosi P, Vitale G (1991). Body position changes redistribute lung computed-tomographic density in patients with acute respiratory failure. Anesthesiology.

[105] Gattinoni L, Pesenti A, Carlesso E (2013). Body position changes redistribute lung computed-tomographic density in patients with acute respiratory failure: impact and clinical fallout through the following 20 years. Intensive Care Med.

[106] Gattinoni L, Tognoni G, Pesenti A (2001). Effect of prone positioning on the survival of patients with acute respiratory failure. N Engl J Med.

[107] Gattinoni L, Vagginelli F, Carlesso E (2003). Decrease in PaCO2 with prone position is predictive of improved outcome in acute respiratory distress syndrome. Crit Care Med.

[108] Gawlinski A, Dracup K (1998). Effect of positioning on SvO2 in the critically ill patient with a low ejection fraction. Nurs Res.

[109] Genc A, Ozyurek S, Koca U (2012). Respiratory and hemodynamic responses to mobilization of critically ill obese patients. Cardiopulm Phys Ther J.

[110] Gentilello L, Thompson DA, Tonnesen AS (1988). Effect of a rotating bed on the incidence of pulmonary complications in critically ill patients. Crit Care Med.

[111] Germann P, Poschl G, Leitner C (1998). Additive effect of nitric oxide inhalation on the oxygenation benefit of the prone position in the adult respiratory distress syndrome. Anesthesiology.

[112] Gerovasili V, Stefanidis K, Vitzilaios K (2009). Electrical muscle stimulation preserves the muscle mass of critically ill patients: a randomized study. Crit Care.

[113] Gianakis A, McNett M, Belle J (2015). Risk factors for ventilator-associated pneumonia: among trauma patients with and without brain injury. J Trauma Nurs.

[114] Gillart T, Bazin JE, Cosserant B (1998). Combined nitric oxide inhalation, prone positioning and almitrine infusion improve oxygenation in severe ARDS. Can J Anaesth.

[115] Gillespie DJ, Rehder K (1987). Body position and ventilation-perfusion relationships in unilateral pulmonary disease. Chest.

[116] Girard R, Baboi L, Ayzac L (2014). The impact of patient positioning on pressure ulcers in patients with severe ARDS: results from a multicentre randomised controlled trial on prone positioning. Intensive Care Med.

[117] Girou E, Buu-Hoi A, Stephan F (2004). Airway colonisation in long-term mechanically ventilated patients. Effect of semi-recumbent position and continuous subglottic suctioning. Intensive Care Med.

[118] Gocze I, Strenge F, Zeman F (2013). The effects of the semirecumbent position on hemodynamic status in patients on invasive mechanical ventilation. Crit Care.

[119] Goettler CE, Pryor JP, Reilly PM (2002). Brachial plexopathy after prone positioning. Crit Care.

[120] Goldhill DR, Imhoff M, McLean B (2007). Rotational bed therapy to prevent and treat respiratory complications: a review and meta-analysis. Am J Crit Care.

[121] Gosselink R, Bott J, Johnson M (2008). Physiotherapy for adult patients with critical illness: recommendations of the European Respiratory Society and European Society of Intensive Care Medicine Task Force on Physiotherapy for Critically Ill Patients. Intensive Care Med.

[122] Grant GP, Szirth BC, Bennett HL (2010). Effects of prone and reverse trendelenburg positioning on ocular parameters. Anesthesiology.

[123] Guerin C (2014). Prone position. Curr Opin Crit Care.

[124] Guerin C, Baboi L, Richard JC (2014). Mechanisms of the effects of prone positioning in acute respiratory distress syndrome. Intensive Care Med.

[125] Guerin C, Badet M, Rosselli S (1999). Effects of prone position on alveolar recruitment and oxygenation in acute lung injury. Intensive Care Med.

[126] Guerin C, Gaillard S, Lemasson S (2004). Effects of systematic prone positioning in hypoxemic acute respiratory failure: a randomized controlled trial. JAMA.

[127] Guerin C, Reignier J, Richard J (2013). Prone positioning in severe acute respiratory distress syndrome. N Engl J Med.

[128] Gui D, Tazza L, Boldrini G (1982). Effects of supine versus sitting bedrest upon blood gas tensions, cardiac output, venous admixture and ventilation-perfusion ratio in man after upper abdominal surgery. Int J Tissue React.

[129] Halm MA (2012). Trendelenburg position: “put to bed” or angled toward use in your unit?. Am J Crit Care.

[130] Hanekom S, Gosselink R, Dean E (2011). The development of a clinical management algorithm for early physical activity and mobilization of critically ill patients: synthesis of evidence and expert opinion and its translation into practice. Clin Rehabil.

[131] Hanekom SD, Louw Q, Coetzee A (2012). The way in which a physiotherapy service is structured can improve patient outcome from a surgical intensive care: a controlled clinical trial. Crit Care.

[132] Hasan FM, Beller TA, Sobonya RE (1982). Effect of positive end-expiratory pressure and body position in unilateral lung injury. J Appl Physiol Respir Environ Exerc Physiol.

[133] Hasegawa I, Boiselle PM, Raptopoulos V (2003). Tracheomalacia incidentally detected on CT pulmonary angiography of patients with suspected pulmonary embolism. Am J Roentgenol.

[134] Hering R, Vorwerk R, Wrigge H (2002). Prone positioning, systemic hemodynamics, hepatic indocyanine green kinetics, and gastric intramucosal energy balance in patients with acute lung injury. Intensive Care Med.

[135] Hering R, Wrigge H, Vorwerk R (2001). The effects of prone positioning on intraabdominal pressure and cardiovascular and renal function in patients with acute lung injury. Anesth Analg.

[136] Hess DR (2005). Patient positioning and ventilator-associated pneumonia. Respir Care.

[137] Hojlund J, Sandmand M, Sonne M (2012). Effect of head rotation on cerebral blood velocity in the prone position. Anesthesiol Res Pract.

[138] Holden J, Dawson J, Horsfield C (2000). Proning patients in intensive care. Nurs Crit Care.

[139] Hopkins RO, Spuhler VJ, Thomsen GE (2007). Transforming ICU culture to facilitate early mobility. Crit Care Clin.

[140] Hoste, Eric AJ, Roosens, Carl DVK, Bracke S (2005). Acute effects of upright position on gas exchange in patients with acute respiratory distress syndrome. J Intensive Care Med.

[141] Hough A (1984). The effect of posture on lung function. Physiotherapy.

[142] Ibanez J, Penafiel A, Raurich JM (1992). Gastroesophageal reflux in intubated patients receiving enteral nutrition: effect of supine and semirecumbent positions. J Parenter Enteral Nutr.

[143] Ibanez J, Raurich JM, Abizanda R (1981). The effect of lateral positions on gas exchange in patients with unilateral lung disease during mechanical ventilation. Intensive Care Med.

[144] Johannigman JA, Davis K, Miller SL (2000). Prone positioning for acute respiratory distress syndrome in the surgical intensive care unit: who, when, and how long?. Surgery.

[145] Johannigman JA, Davis K, Miller SL (2001). Prone positioning and inhaled nitric oxide: synergistic therapies for acute respiratory distress syndrome. J Trauma.

[146] Jolliet P, Bulpa P, Chevrolet JC (1998). Effects of the prone position on gas exchange and hemodynamics in severe acute respiratory distress syndrome. Crit Care Med.

[147] Jones AT, Hansell DM, Evans TW (2001). Pulmonary perfusion in supine and prone positions: an electron-beam computed tomography study. J Appl Physiol.

[148] Jones, Alice YM, Dean E (2004). Body position change and its effect on hemodynamic and metabolic status. Heart Lung.

[149] Jozwiak M, Teboul J, Anguel N (2013). Beneficial Hemodynamic Effects of Prone Positioning in Patients with Acute Respiratory Distress Syndrome. Am J Respir Crit Care Med.

[150] Kalisch BJ, Dabney BW, Lee S (2013). Safety of mobilizing hospitalized adults: review of the literature. J Nurs Care Qual.

[151] Karatzanos E, Gerovasili V, Zervakis D (2012). Electrical muscle stimulation: an effective form of exercise and early mobilization to preserve muscle strength in critically ill patients. Crit Care Res Pract.

[152] Kasotakis G, Schmidt U, Perry D (2012). The surgical intensive care unit optimal mobility score predicts mortality and length of stay. Crit Care Med.

[153] Kayambu G, Boots R, Paratz J (2013). Physical therapy for the critically ill in the ICU: a systematic review and meta-analysis. Crit Care Med.

[154] Keeley L (2007). Reducing the risk of ventilator-acquired pneumonia through head of bed elevation. Nurs Crit Care.

[155] Kenning JA, Toutant SM, Saunders RL (1981). Upright patient positioning in the management of intracranial hypertension. Surg Neurol.

[156] de Keulenaer BL, de Waele JJ, Powell B (2009). What is normal intra-abdominal pressure and how is it affected by positioning, body mass and positive end-expiratory pressure?. Intensive Care Med.

[157] Kiefer P, Morin A, Putzke C (2001). Influence of prone position on gastric mucosal-arterial PCO2 gradients. Intensive Care Med.

[158] Kipping V, Weber-Carstens S, Lojewski C (2013). Prone position during ECMO is safe and improves oxygenation. Int J Artif Organs.

[159] Kirkeby-Garstad I, Sellevold OFM, Stenseth R (2005). Mixed venous oxygen desaturation during early mobilization after coronary artery bypass surgery. Acta Anaesthesiol Scand.

[160] Kirschenbaum L, Azzi E, Sfeir T (2002). Effect of continuous lateral rotational therapy on the prevalence of ventilator-associated pneumonia in patients requiring long-term ventilatory care. Crit Care Med.

[161] Klingstedt C, Hedenstierna G, Baehrendtz S (1990). Ventilation-perfusion relationships and atelectasis formation in the supine and lateral positions during conventional mechanical and differential ventilation. Acta Anaesthesiol Scand.

[162] Kommission für Krankenhaushygiene und Infektionsprävention (KRINKO) (2013). Prävention der nosokomialen beatmungsassoziierten Pneumonie. Bundesgesundheitsbl.

[163] Kopterides P, Siempos II, Armaganidis A (2009). Prone positioning in hypoxemic respiratory failure: meta-analysis of randomized controlled trials. J Crit Care.

[164] Kredel M, Bischof L, Wurmb T (2013). Combination of positioning therapy and venovenous extracorporeal membrane oxygenation in ARDS patients. Perfusion.

[165] Kurnutala LN, Joshi M, Kamath H (2014). A surprising cause of wheezing in a morbidly obese patient: a case report. Int Med Case Rep J.

[166] Lamm WJ, Graham MM, Albert RK (1994). Mechanism by which the prone position improves oxygenation in acute lung injury. Am J Respir Crit Care Med.

[167] Langer M, Mascheroni D, Marcolin R (1988). The prone position in ARDS patients. A clinical study. Chest.

[168] Leditschke IA, Green M, Irvine J (2012). What are the barriers to mobilizing intensive care patients?. Cardiopulm Phys Ther J.

[169] Lee DL, Chiang H, Lin S (2002). Prone-position ventilation induces sustained improvement in oxygenation in patients with acute respiratory distress syndrome who have a large shunt. Crit Care Med.

[170] Lee K, Kim M, Yoo J (2010). Clinical meaning of early oxygenation improvement in severe acute respiratory distress syndrome under prolonged prone positioning. Korean J Intern Med.

[171] Legras A, Dequin PF, Hazouard E (1999). Right-to-left interatrial shunt in ARDS: dramatic improvement in prone position. Intensive Care Med.

[172] Lemyze M, Guerry M, Mallat J (2012). Obesity supine death syndrome revisited. Eur Respir J.

[173] Lemyze M, Mallat J, Duhamel A (2013). Effects of Sitting Position and Applied Positive End-Expiratory Pressure on Respiratory Mechanics of Critically Ill Obese Patients Receiving Mechanical Ventilation. Crit Care Med.

[174] Lemyze M, Taufour P, Duhamel A (2014). Determinants of noninvasive ventilation success or failure in morbidly obese in patients in acute respiratory failure. Plos One.

[175] L E, Renault A, Oger E (2002). A prospective survey of early 12-h prone positioning effects in patients with the acute respiratory distress syndrome. Intensive Care Med.

[176] Li Z, Peng X, Zhu B (2013). Active mobilization for mechanically ventilated patients: a systematic review. Arch Phys Med Rehab.

[177] Lim CM, Kim EK, Lee JS (2001). Comparison of the response to the prone position between pulmonary and extrapulmonary acute respiratory distress syndrome. Intensive Care Med.

[178] Linn DD, Beckett RD, Foellinger K (2014). Administration of enteral nutrition to adult patients in the prone position. Intensive Crit Care Nurs.

[179] Lipshutz AKM, Gropper MA (2013). Acquired neuromuscular weakness and early mobilization in the intensive care unit. Anesthesiology.

[180] Lodrini S, Montolivo M, Pluchino F (1989). Positive end-expiratory pressure in supine and sitting positions: its effects on intrathoracic and intracranial pressures. Neurosurgery.

[181] Lord RK, Mayhew CR, Korupolu R (2013). ICU early physical rehabilitation programs: financial modeling of cost savings. Crit Care Med.

[182] Lyerla F, LeRouge C, Cooke DA (2010). A nursing clinical decision support system and potential predictors of head-of-bed position for patients receiving mechanical ventilation. Am J Crit Care.

[183] Mackenzie CF (2001). Anatomy, physiology, and pathology of the prone position and postural drainage. Crit Care Med.

[184] Maclntyre NR, Helms M, Wunderink R (1999). Automated rotational therapy for the prevention of respiratory complications during mechanical ventilation. Respir Care.

[185] Mancebo J, Fernandez R, Blanch L (2006). A multicenter trial of prolonged prone ventilation in severe acute respiratory distress syndrome. Am J Respir Crit Care Med.

[186] Martinez M, Diaz E, Joseph D (1999). Improvement in oxygenation by prone position and nitric oxide in patients with acute respiratory distress syndrome. Intensive Care Med.

[187] Matejovic M, Rokyta R, Radermacher P (2002). Effect of prone position on hepato-splanchnic hemodynamics in acute lung injury. Intensive Care Med.

[188] McAuley DF, Giles S, Fichter H (2002). What is the optimal duration of ventilation in the prone position in acute lung injury and acute respiratory distress syndrome?. Intensive Care Med.

[189] McBeth PB, Zygun DA, Widder S (2007). Effect of patient positioning on intra-abdominal pressure monitoring. Am J Surg.

[190] McCormick J, Blackwood B (2001). Nursing the ARDS patient in the prone position: the experience of qualified ICU nurses. Intensive Crit Care Nurs.

[191] Meininger D, Zwissler B, Byhahn C (2006). Impact of overweight and pneumoperitoneum on hemodynamics and oxygenation during prolonged laparoscopic surgery. World J Surg.

[192] Mendez-Tellez PA, Needham DM (2012). Early physical rehabilitation in the ICU and ventilator liberation. Respir Care.

[193] Mentzelopoulos SD, Roussos C, Zakynthinos SG (2005). Prone position reduces lung stress and strain in severe acute respiratory distress syndrome. Eur Respir J.

[194] Mentzelopoulos SD, Sigala J, Roussos C (2006). Static pressure–volume curves and body posture in severe chronic bronchitis. Eur Respir J.

[195] Messerole E, Peine P, Wittkopp S (2002). The pragmatics of prone positioning. Am J Respir Crit Care Med.

[196] Meyer W (1914). Der Siegeszug der Beckenhochlagerung. Deutsche Z Chirurgie.

[197] Meyer W (1885). Ueber die Nachbehandlung des hohen Steinschnittes sowie uber Verwendbarkeit desselben zur Operation von Blasenscheidenfisteln. Archiv für Chirurgie.

[198] Michelet P, Roch A, Gainnier M (2005). Influence of support on intra-abdominal pressure, hepatic kinetics of indocyanine green and extravascular lung water during prone positioning in patients with ARDS: a randomized crossover study. Crit Care.

[199] Morris PE, Goad A, Thompson C (2008). Early intensive care unit mobility therapy in the treatment of acute respiratory failure. Crit Care Med.

[200] Mounier R, Adrie C, Francais A (2010). Study of prone positioning to reduce ventilator-associated pneumonia in hypoxaemic patients. Eur Respir J.

[201] Mulamula A, Winter SM, Schweizer K et al (2010) Implementation of a program of early mobilization of icu patients without additional staff resources using a capacity assessment and exercise prescription tool and culture change. Am J Respir Crit Care Med 181: A6717

[202] Mullins CD, Philbeck TE, Schroeder WJ, Thomas SK (2002). Cost effectiveness of kinetic therapy in preventing nosocomial lower respiratory tract infections in patients suffering from trauma. Manag Care Interface.

[203] Mure M, Lindahl SG (2001). Prone position improves gas exchange–but how?. Acta Anaesthesiol Scand.

[204] Mure M, Domino KB, Lindahl SG (2000). Regional ventilation-perfusion distribution is more uniform in the prone position. J Appl Physiol.

[205] Mure M, Glenny RW, Domino KB (1998). Pulmonary gas exchange improves in the prone position with abdominal distension. Am J Respir Crit Care Med.

[206] Mure M, Martling CR, Lindahl SG (1997). Dramatic effect on oxygenation in patients with severe acute lung insufficiency treated in the prone position. Crit Care Med.

[207] Murray TA, Patterson LA (2002). Prone positioning of trauma patients with acute respiratory distress syndrome and open abdominal incisions. Crit Care Nurse.

[208] Nava S (1998). Rehabilitation of patients admitted to a respiratory intensive care unit. Arch Phys Med Rehab.

[209] Nekludov M, Bellander B, Mure M (2006). Oxygenation and cerebral perfusion pressure improved in the prone position. Acta Anaesthesiol Scand.

[210] Nelson LD, Anderson HB (1989). Physiologic effects of steep positioning in the surgical intensive care unit. Arch Surg.

[211] Nelson LD, Choi SC (1992). Kinetic therapy in critically ill trauma patients. Clin Intensive Care.

[212] Nielsen KG, Holte K, Kehlet H (2003). Effects of posture on postoperative pulmonary function. Acta Anaesthesiol Scand.

[213] Niël-Weise BS (2011). An evidence-based recommendation on bed head elevation for mechanically ventilated patients. Critical Care.

[214] van Nieuwenhoven CA, Vandenbroucke-Grauls C, van Tiel FH (2006). Feasibility and effects of the semirecumbent position to prevent ventilator-associated pneumonia: a randomized study. Crit Care Med.

[215] Nyren S, Mure M, Jacobsson H (1999). Pulmonary perfusion is more uniform in the prone than in the supine position: scintigraphy in healthy humans. J Appl Physiol.

[216] Nyren S, Radell P, Lindahl SGE (2010). Lung ventilation and perfusion in prone and supine postures with reference to anesthetized and mechanically ventilated healthy volunteers. Anesthesiology.

[217] Offner PJ, Haenel JB, Moore EE (2000). Complications of prone ventilation in patients with multisystem trauma with fulminant acute respiratory distress syndrome. J Trauma.

[218] Olkowski BF, Devine MA, Slotnick LE (2013). Safety and feasibility of an early mobilization program for patients with aneurysmal subarachnoid hemorrhage. Phys Ther.

[219] Orozco-Levi M, Torres A, Ferrer M (1995). Semirecumbent position protects from pulmonary aspiration but not completely from gastroesophageal reflux in mechanically ventilated patients. Am J Respir Crit Care Med.

[220] Papazian L, Bregeon F, Gaillat F (1998). Respective and combined effects of prone position and inhaled nitric oxide in patients with acute respiratory distress syndrome. Am J Respir Crit Care Med.

[221] Papazian L, Paladini M, Bregeon F (2002). Can the tomographic aspect characteristics of patients presenting with acute respiratory distress syndrome predict improvement in oxygenation-related response to the prone position?. Anesthesiology.

[222] Pape HC, Regel G, Borgmann W (1993). Effect of continuous change in axial position in treatment of post-traumatic lung failure (ARDS). A clinical study. Unfallchirurgie.

[223] Pape HC, Regel G, Borgmann W (1994). The effect of kinetic positioning on lung function and pulmonary haemodynamics in posttraumatic ARDS: a clinical study. Injury.

[224] Pape HC, Remmers D, Weinberg A (1998). Is early kinetic positioning beneficial for pulmonary function in multiple trauma patients?. Injury.

[225] Pappert D, Rossaint R, Slama K (1994). Influence of positioning on ventilation-perfusion relationships in severe adult respiratory distress syndrome. Chest.

[226] Pedersen T, Moller AM (2001). How to use evidence-based medicine in anaesthesiology (review). Acta Anaesthesiol Scand.

[227] Pelosi P, Bottino N, Chiumello D (2003). Sigh in supine and prone position during acute respiratory distress syndrome. Am J Respir Crit Care Med.

[228] Pelosi P, Brazzi L, Gattinoni L (2002). Prone position in acute respiratory distress syndrome. Eur Respir J.

[229] Pelosi P, Tubiolo D, Mascheroni D (1998). Effects of the prone position on respiratory mechanics and gas exchange during acute lung injury. Am J Respir Crit Care Med.

[230] Perme C, Chandrashekar R (2009). Early mobility and walking program for patients in intensive care units: creating a standard of care. Am J Crit Care.

[231] Peterlini MAS, Rocha PK, Kusahara DM (2006). Subjective assessment of backrest elevation: magnitude of error. Heart Lung.

[232] Peterson M, Schwab W, McCutcheon K (2008). Effects of elevating the head of bed on interface pressure in volunteers. Crit Care Med.

[233] Porta R, Vitacca M, Clini E (1999). Physiological effects of posture on mask ventilation in awake stable chronic hypercapnic COPD patients. Eur Respir J.

[234] Prebio M, Katz-Papatheophilou E, Heindl W (2005). Reduction of pressure sores during prone positioning of ventilated intensive care patients by the prone-head support system: a pilot study. Wien Klin Wochenschr.

[235] Prokocimer P, Garbino J, Wolff M (1983). Influence of posture on gas exchange in artificially ventilated patients with focal lung disease. Intensive Care Med.

[236] Protti A, Chiumello D, Cressoni M (2009). Relationship between gas exchange response to prone position and lung recruitability during acute respiratory failure. Intensive Care Med.

[237] Rance M (2005). Kinetic therapy positively influences oxygenation in patients with ALI/ARDS. Nurs Crit Care.

[238] Raoof S, Chowdhrey N, Feuerman M (1999). Effect of combined kinetic therapy and percussion therapy on the resolution of atelectasis in critically ill patients. Chest.

[239] Rehder K (1998). Postural changes in respiratory function. Acta Anaesthesiol Scand Suppl.

[240] Reignier J, Thenoz-Jost N, Fiancette M (2004). Early enteral nutrition in mechanically ventilated patients in the prone position. Crit Care Med.

[241] Reinprecht A, Greher M, Wolfsberger S (2003). Prone position in subarachnoid hemorrhage patients with acute respiratory distress syndrome: effects on cerebral tissue oxygenation and intracranial pressure. Crit Care Med.

[242] Reutershan J, Schmitt A, Dietz K (2006). Alveolar recruitment during prone position: time matters. Clin Sci.

[243] Rialp G, Betbese AJ, Perez-Marquez M (2001). Short-term effects of inhaled nitric oxide and prone position in pulmonary and extrapulmonary acute respiratory distress syndrome. Am J Respir Crit Care Med.

[244] Rivara D, Artucio H, Arcos J (1984). Positional hypoxemia during artificial ventilation. Crit Care Med.

[245] Robak O, Schellongowski P, Bojic A (2011). Short-term effects of combining upright and prone positions in patients wi th ARDS: a prospective randomized study. Critical Care.

[246] Rodriguez PO, Setten M, Maskin LP (2012). Muscle weakness in septic patients requiring mechanical ventilation: protective effect of transcutaneous neuromuscular electrical stimulation. J Crit Care.

[247] Rooban N, Regli A, Davis WA (2012). Comparing intra-abdominal pressures in different body positions via a urinary catheter and nasogastric tube: a pilot study. Ann Intensive Care.

[248] Rose L, Baldwin I, Crawford T (2009). Recumbency with continuous measurement using beddials, education and teamwork (Recumbent). Crit Care Med.

[249] Rose L, Baldwin I, Crawford T (2010). Semirecumbent positioning in ventilator-dependent patients: a multicenter, observational study. Am J Crit Care.

[250] Rose L, Baldwin I, Crawford T (2010). The use of bed-dials to maintain recumbent positioning for critically ill mechanically ventilated patients (The RECUMBENT study): multicentre before and after observational study. Int J Nurs Stud.

[251] Rosner MJ, Coley IB (1986). Cerebral perfusion pressure, intracranial pressure, and head elevation. J Neurosurg.

[252] Ross DJ, Wu P, Mohsenifar Z (1997). Assessment of postural differences in regional pulmonary perfusion in man by single-photon emission computerized tomography. Clin Sci.

[253] Routsi C, Gerovasili V, Vasileiadis I (2010). Electrical muscle stimulation prevents critical illness polyneuromyopathy: a randomized parallel intervention trial. Crit Care.

[254] Rowe C (2004). Development of clinical guidelines for prone positioning in critically ill adults. Nurs Crit Care.

[255] Saez de la Fuente I, Saez de la Fuente J, Quintana Estelles MD (2014). Enteral Nutrition in Patients Receiving Mechanical Ventilation in a Prone Position. J Parent Ent Nutr.

[256] Schellongowski P, Losert H, Locker GJ (2007). Prolonged lateral steep position impairs respiratory mechanics during continuous lateral rotation therapy in respiratory failure. Intensive Care Med.

[257] Schmitz TM (1991). The semi-prone position in ARDS: five case studies. Crit Care Nurse.

[258] Schweickert WD, Kress JP (2011). Implementing Early Mobilization Interventions in Mechanically Ventilated Patients in the ICU. Chest.

[259] Schweickert WD, Pohlman MC, Pohlman AS (2009). Early physical and occupational therapy in mechanically ventilated, critic ally ill patients: a randomised controlled trial. Lancet.

[260] Seaton-Mills D (2000). Prone positioning in ARDS: a nursing perspective. Clin Intensive Care.

[261] Servillo G, Roupie E, de Robertis E (1997). Effects of ventilation in ventral decubitus position on respiratory mechanics in adult respiratory distress syndrome. Intensive Care Med.

[262] Shuster MH, Sekula LK, Kern JC (2011). Measuring intrabladder pressure with the head of the bed elevated 30 degrees. Evidence to support a change in practice. Am J Crit Care.

[263] Simonis G, Steiding K, Schaefer K (2012). A prospective, randomized trial of continuous lateral rotation (“kinetic therapy”) in patients with cardiogenic shock. Clin Res Cardiol.

[264] Sprung J, Whalley DG, Falcone T (2003). The effects of tidal volume and respiratory rate on oxygenation and respiratory mechanics during laparoscopy in morbidly obese patients. Anesth Analg.

[265] Staudinger T, Bojic A, Holzinger U (2010). Continuous lateral rotation therapy to prevent ventilator-associated pneumonia. Crit Care Med.

[266] Staudinger T, Kofler J, Mullner M (2001). Comparison of prone positioning and continuous rotation of patients with adult respiratory distress syndrome: results of a pilot study. Crit Care Med.

[267] Stiletto R, Gotzen L, Goubeaud S (2000). Kinetic therapy for therapy and prevention of post-traumatic lung failure. Results of a prospective study of 111 polytrauma patients. Unfallchirurg.

[268] Stiller K (2007). Safety issues that should be considered when mobilizing critically ill patients. Crit Care Clin.

[269] Stiller K (2013). Physiotherapy in intensive care: an updated systematic review. Chest.

[270] Stiller K, Phillips AC, Lambert P (2004). The safety of mobilisation and its effect on haemodynamic and respiratory status of intensive care patients. Physiother Theory Pract.

[271] Stocker R, Neff T, Stein S (1997). Prone postioning and low-volume pressure-limited ventilation improve survival in patients with severe ARDS. Chest.

[272] Sud S, Friedrich JO, Adhikari NK, Mancebo J (2014). Effect of prone positioning during mechanical ventilation on mortality among patients with acute respiratory distress syndrome: a systematic review and meta-analysis. CMAJ.

[273] Sud S, Friedrich JO, Taccone P (2010). Prone ventilation reduces mortality in patients with acute respiratory failure and severe hypoxemia: systematic review and meta-analysis. Intensive Care Med.

[274] Sud S, Sud M, Friedrich JO (2008). Effect of mechanical ventilation in the prone position on clinical outcomes in patients with acute hypoxemic respiratory failure: a systematic review and meta-analysis. CMAJ.

[275] Sud S, Sud M, Friedrich JO (2008). Effect of prone positioning in patients with acute respiratory distress syndrome and high Simplified Acute Physiology Score II. Crit Care Med.

[276] Sullivan J (2000). Positioning of patients with severe traumatic brain injury: research-based practice. J Neurosci Nurs.

[277] Summer WR, Curry P, Haponik EF (1989). Continuous mechanical turning of intensive care unit patients shortens length of stay in some diagnostic-related groups. J Crit Care.

[278] Suter PM (2006). Reducing ventilator-induced lung injury and other organ injury by the prone position. Crit Care.

[279] Swadener-Culpepper L, Skaggs RL, Vangilder CA (2008). The impact of continuous lateral rotation therapy in overall clinical and financial outcomes of critically ill patients. Crit Care Nurs Q.

[280] Taccone P, Pesenti A, Latini R (2009). Prone positioning in patients with moderate and severe acute respiratory d istress syndrome: a randomized controlled trial. JAMA.

[281] Talley CL, Wonnacott RO, Schuette JK (2013). Extending the benefits of early mobility to critically ill patients undergoing continuous renal replacement therapy: the Michigan experience. Crit Care Nurs Q.

[282] Tanskanen P, Kytta J, Randell T (1997). The effect of patient positioning on dynamic lung compliance. Acta Anaesthesiol Scand.

[283] Thelandersson A, Cider A, Nellgard B (2006). Prone position in mechanically ventilated patients with reduced intracranial compliance. Acta Anaesthesiol Scand.

[284] Thomas AR, Bryce TL (1998). Ventilation in the patient with unilateral lung disease. Crit Care Clin.

[285] Thomas PJ, Paratz JD (2007). Is there evidence to support the use of lateral positioning in intensive care? A systematic review. Anaesth Intensive Care.

[286] Thomas PJ, Paratz JD, Lipman J (2007). Lateral positioning of ventilated intensive care patients: a study of oxygenation, respiratory mechanics, hemodynamics, and adverse events. Heart Lung.

[287] Tillett JM, Marmarou A, Agnew JP (1993). Effect of continuous rotational therapy on intracranial pressure in the severely brain-injured patient. Crit Care Med.

[288] Tiruvoipati R, Bangash M, Manktelow B (2008). Efficacy of prone ventilation in adult patients with acute respiratory failure: a meta-analysis. J Crit Care.

[289] Titsworth WL, Hester J, Correia T (2012). The effect of increased mobility on morbidity in the neurointensive care unit. J Neurosurg Spine.

[290] Torres A, Serra-Batlles J, Ros E (1992). Pulmonary aspiration of gastric contents in patients receiving mechanical ventilation: the effect of body position. Ann Intern Med.

[291] Traver GA, Tyler ML, Hudson LD (1995). Continuous oscillation: outcome in critically ill patients. J Crit Care.

[292] Tsueda K, Debrand M, Zeok SS (1979). Obesity supine death syndrome: reports of two morbidly obese patients. Anesth Analg.

[293] Valenza F, Guglielmi M, Maffioletti M (2005). Prone position delays the progression of ventilator-induced lung injury in rats: does lung strain distribution play a role?. Crit Care Med.

[294] van der Voort PH, Zandstra DF (2001). Enteral feeding in the critically ill: comparison between the supine and prone positions: a prospective crossover study in mechanically ventilated patients. Crit Care.

[295] Varpula T, Jousela I, Niemi R (2003). Combined effects of prone positioning and airway pressure release ventilation on gas exchange in patients with acute lung injury. Acta Anaesthesiol Scand.

[296] Vasquez DG, Berg-Copas GM, Wetta-Hall R (2007). Influence of semi-recumbent position on intra-abdominal pressure as measured by bladder pressure. J Surg Res.

[297] Venet C, Guyomarc S, Pingat J (2003). Prognostic factors in acute respiratory distress syndrome: a retrospective multivariate analysis including prone positioning in management strategy. Intensive Care Med.

[298] Vieillard-Baron A, Charron C, Caille V (2007). Prone positioning unloads the right ventricle in severe ARDS. Chest.

[299] Vieillard-Baron A, Rabiller A, Chergui K (2005). Prone position improves mechanics and alveolar ventilation in acute respiratory distress syndrome. Intensive Care Med.

[300] Voggenreiter G, Aufmkolk M, Stiletto RJ (2005). Prone positioning improves oxygenation in post-traumatic lung injury–a prospective randomized trial. J Trauma.

[301] Voggenreiter G, Neudeck F, Aufmkolk M (1999). Intermittent prone positioning in the treatment of severe and moderate posttraumatic lung injury. Crit Care Med.

[302] Voggenreiter G, Neudeck F, Obertacke U (1995). Intermittent dorso-ventral positioning in therapy of severe post-traumatic lung failure. Unfallchirurg.

[303] Vollman KM (1997). Prone positioning for the ARDS patient. Dimens Crit Care Nurs.

[304] Vollman KM (2004). Prone positioning in the patient who has acute respiratory distress syndrome: the art and science. Crit Care Nurs Clin North Am.

[305] Wageck B, Nunes GS, Silva FL (2014). Application and effects of neuromuscular electrical stimulation in critically ill patients: systematic review. Med Intensiva.

[306] Ward NS (2002). Effects of prone position ventilation in ARDS. An evidence-based review of the literature. Crit Care Clin.

[307] Watanabe I, Fujihara H, Sato K (2002). Beneficial effect of a prone position for patients with hypoxemia after transthoracic esophagectomy. Crit Care Med.

[308] Weber-Carstens S, Schneider J, Wollersheim T (2013). Critical illness myopathy and GLUT4: significance of insulin and muscle contraction. Am J Respir Crit Care Med.

[309] Weig T, Janitza S, Zoller M (2014). Influence of abdominal obesity on multiorgan dysfunction and mortality in acute respiratory distress syndrome patients treated with prone positioning. J Crit Care.

[310] Whiteman K, Nachtmann L, Kramer D (1995). Effects of continuous lateral rotation therapy on pulmonary complications in liver transplant patients. Am J Crit Care.

[311] Williams Z, Chan R, Kelly E (2008). A simple device to increase rates of compliance in maintaining 30-degree head-of-bed elevation in ventilated patients. Crit Care Med.

[312] Wilson AE, Bermingham-Mitchell K, Wells N (1996). Effect of backrest position on hemodynamic and right ventricular measurements in critically ill adults. Am J Crit Care.

[313] Winkelman C (2000). Effect of backrest position on intracranial and cerebral perfusion pressures in traumatically brain-injured adults. Am J Crit Care.

[314] Wolken RF, Woodruff RJ, Smith J (2012). Observational study of head of bed elevation adherence using a continuous monitoring system in a medical intensive care unit. Respir Care.

[315] Yamaguchi S, Hirakawa K, Kitamura J (2013). A case of ventilation disorder and poor oxygenation after changing position from prone to supine. Masui.

[316] Yanase LR, Baraban E, Stuchiner TL (2013). A safe early mobility protocol for stroke patients. Stroke.

[317] Zanni JM, Korupolu R, Fan E (2010). Rehabilitation therapy and outcomes in acute respiratory failure: an observational pilot project. J Crit Care.

[318] Zeppos L, Patman S, Berney S (2007). Physiotherapy in intensive care is safe: an observational study. Aust J Physiother.

[319] Zomorodi M, Topley D, McAnaw M (2012). Developing a mobility protocol for early mobilization of patients in a surgical/trauma ICU. Crit Care Res Pract.

